# *Pot1b* ^−/−^ tumors activate G-quadruplex-induced DNA damage to promote telomere hyper-elongation

**DOI:** 10.1093/nar/gkad648

**Published:** 2023-08-10

**Authors:** Taylor Takasugi, Peili Gu, Fengshan Liang, Isabelle Staco, Sandy Chang

**Affiliations:** Department of Laboratory Medicine, Yale University School of Medicine, New Haven, CT 06520, USA; Department of Laboratory Medicine, Yale University School of Medicine, New Haven, CT 06520, USA; Department of Laboratory Medicine, Yale University School of Medicine, New Haven, CT 06520, USA; Department of Laboratory Medicine, Yale University School of Medicine, New Haven, CT 06520, USA; Department of Laboratory Medicine, Yale University School of Medicine, New Haven, CT 06520, USA; Department of Molecular Biophysics and Biochemistry, Yale University School of Medicine, New Haven, CT 06520, USA; Department of Pathology, Yale University School of Medicine, New Haven, CT 06520, USA

## Abstract

Malignant cancers must activate telomere maintenance mechanisms to achieve replicative immortality. Mutations in the human Protection of Telomeres 1 (*POT1*) gene are frequently detected in cancers with abnormally long telomeres, suggesting that the loss of POT1 function disrupts the regulation of telomere length homeostasis to promote telomere elongation. However, our understanding of the mechanisms leading to elongated telomeres remains incomplete. The mouse genome encodes two POT1 proteins, POT1a and POT1b possessing separation of hPOT1 functions. We performed serial transplantation of *Pot1b^−/−^* sarcomas to better understand the role of POT1b in regulating telomere length maintenance. While early-generation *Pot1b^−/−^* sarcomas initially possessed shortened telomeres, late-generation *Pot1b^−/−^* cells display markedly hyper-elongated telomeres that were recognized as damaged DNA by the Replication Protein A (RPA) complex. The RPA-ATR-dependent DNA damage response at telomeres promotes telomerase recruitment to facilitate telomere hyper-elongation. POT1b, but not POT1a, was able to unfold G-quadruplex present in hyper-elongated telomeres to repress the DNA damage response. Our findings demonstrate that the repression of the RPA-ATR DDR is conserved between POT1b and human POT1, suggesting that similar mechanisms may underly the phenotypes observed in human cancers harboring human POT1 mutations.

## Graphical Abstract

**Graphical Abstract:**
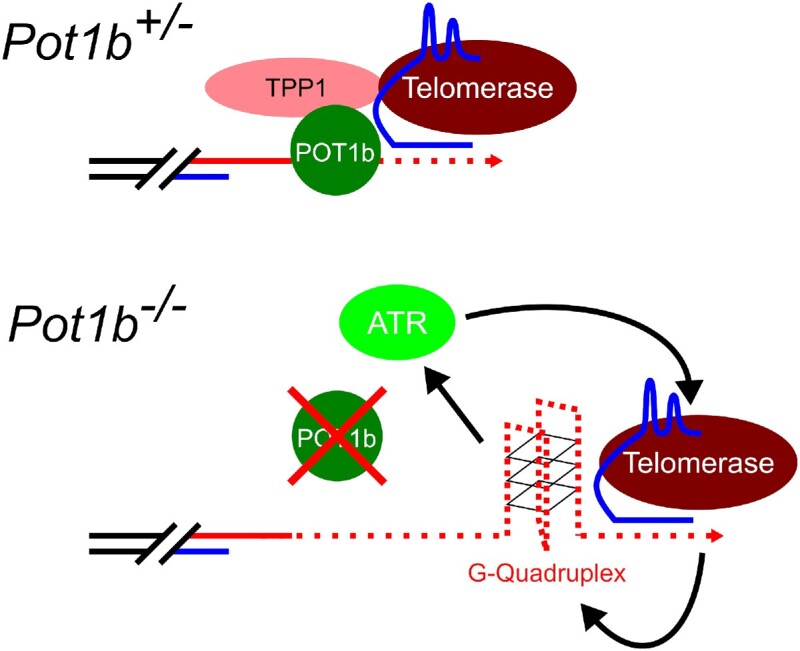

## INTRODUCTION

Telomeres are DNA-protein complexes that cap the ends of eukaryotic linear chromosomes (1,2). Mammalian telomeres are composed of mostly duplex 5′-TTAGGG-3′ DNA hexameric repeats ending in a 3′ single-stranded overhang. Due to the end replication problem, telomeres shorten with each round of cell division, ultimately leading to activation of p53-dependent replicative senescence (3,4). In stem cells and most cancers, telomeres are elongated by the ribonucleoprotein complex telomerase, composed of the telomerase reverse transcriptase (TERT) catalytic component and the RNA templating component (*Terc*) (5–7). The shelterin complex, comprised of six specialized proteins, mediates telomere end protection by repressing the activation of DNA damage response (DDR) pathways. TRF1 (Telomeric repeat-binding factor 1) and TRF2 (Telomeric repeat-binding factor 2) bind to the double-stranded telomeric DNA, while POT1 (Protection of telomeres 1) binds to the single-stranded (ss) telomeric DNA and interacts with TPP1 (Adrenocortical dysplasia protein homolog). TIN2 (TRF1-interacting nuclear protein 2) bridges TPP1-POT1 with TRF1 and TRF2. POT1 forms a functional heterodimer with TPP1, and in turn, TPP1 tethers POT1 to telomeres by interacting with the TIN2-TRF1 and TIN2-TRF2 complexes (1).

POT1 homologs have been identified in all eukaryotes. While most vertebrates, including humans, possess a single *POT1* gene, the rodent genome encodes two *Pot1* genes, *Pot1a* and *Pot1b*, due to recent gene duplication (8–10). All POT1 proteins contain two highly conserved oligosaccharide-oligonucleotide (OB) folds that interact with the 3′ terminus of the telomere ss DNA overhang to exert telomere end protection (9–12). We and others discovered that POT1a and POT1b possess separate functions at telomeres. POT1a protects telomeres from being recognized as damaged DNA by binding to the telomere ss DNA, preventing Replication protein A (RPA) access and the initiation of an ATR-CHK1 mediated DDR signaling (11–15). POT1b binds telomere ss DNA as robustly as POT1a, but it is unclear why POT1b does not play a major role in telomere end protection. Rather, POT1b is involved in the formation of the 3′ ss telomere overhang (16). POT1b also recruits the CTC1-STN1-TEN1 (CST) complex to promote DNA Polymerase-α fill-in synthesis of the C-strand and modulates exonuclease activities at the C-strand (17–20). Consequently, deletion of POT1b in mice increases G-overhang length while accelerating overall telomere shortening (21,22). We discovered recently that POT1b, but not POT1a, plays important roles in promoting telomerase recruitment to telomeres to extend the G-overhang (23). This result suggests that human POT1 (hPOT1) might also be involved in the regulation of telomere length. Indeed, hPOT1 and hTPP1 recruit the CST-complex to telomeres to regulate telomerase activity at the G-strand and promotes fill-in synthesis of the telomeric C-strand (16,19,24).

Next generation sequencing of a wide variety of cancers, including melanoma, chronic lymphocytic leukemia, angiosarcomas and gliomas, has identified recurrent somatic mutations in hPOT1, suggesting that hPOT1 functions as an important tumor suppressor (25,26). Many hPOT1 cancer mutations cluster in its OB folds to disrupt binding to ss-telomeric DNA (27–32). One of the most striking phenotypes observed in patients bearing hPOT1 cancer mutations is increased telomere elongation, likely an important cancer promoting mechanism (28–31,33–37). Recent data on a population of hPOT1-mutant carriers reveal that the long telomeres generated sustain clonal evolution to promote clonal hematopoiesis (38). Sustained clonal evolution allows for the accumulation of additional pro-oncogenic mutations to allow initiated pre-cancer cells to escape replicative senescence in favor of tumor progression. While telomere elongation may explain why hPOT1 mutations promote oncogenesis, our understanding of how hPOT1 dysfunction leads to the generation of these long telomeres remains incomplete. While inhibition of the DDR can repress the telomere elongation observed in hPOT1 OB mutants, telomere elongation has also been observed in tumors bearing hPOT1 cancer mutations without overt DDR activation (34,39).

The two important functions of hPOT1, repression of ATR-DDR activation and regulation of telomere length, are split between mouse POT1a and POT1b respectively. In this study, we utilized these ‘separation-of-functions’ POT1 mouse models to better understand how cancer mutations in hPOT1 promote telomere elongation. We generated *Pot1b^−/−^* sarcomas and extensively passaged them through serial transplantation in the flanks of SCID mice. While telomere lengths in these tumors initially shortened, we found that the extensive passaging of these tumors eventually led to telomere hyper-elongation to lengths that exceeded those observed in *Pot1b^+/−^* cells. We show that late-generation *Pot1b^−/−^* sarcoma telomeres contain G-quadruplexes, which activate an ATR-dependent DDR to recruit telomerase to hyper-elongate telomeres. Increased G-quadruplex accumulation in the telomeric overhang would normally be resolved by POT1b or hPOT1 but not by POT1a. Our results uncover a unique DNA damage protective role for POT1b and provide mechanistic insights into how hPOT1 regulates telomere length.

## MATERIALS AND METHODS

### Generation of MEFs and sarcoma cell lines

**Figure 1.**
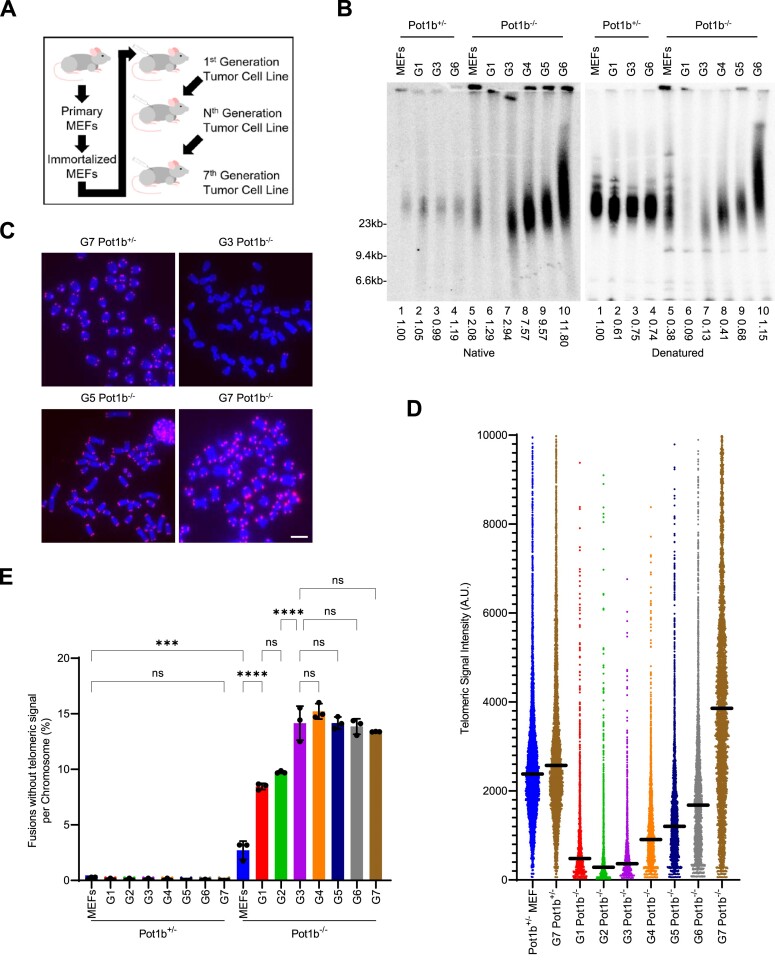
Telomere hyper-elongation in late-generation *Pot1b^−/−^* cells. (**A**) Schematic of serial transplantation through subcutaneous injections in SCID mice. (**B**) TRF Southern blot detection of G-overhang in native gel and total telomere length in denatured gel hybridization with γ-^32^P-(CCCTAA)_4_ telomere probe. Numbers indicate relative G-overhang and total telomere signals, with telomere signals set to 1.0 for Pot1b^+/−^ MEFs. Molecular weight markers as indicated. (**C**) Representative images of metaphase spreads of the indicated cell lines visualized with PNA-FISH probe Cy3-OO-(CCCTAA)_3_ and DAPI. Scale bar: 5μm. (**D**) Q-FISH analysis showing the median telomeric signal intensity from metaphases in (C). 30 metaphases were scored per cell line. (**E**) Quantification of chromosomal fusions in metaphases in (C). Data show the mean ± standard deviation from three independent experiments with at least 1000 chromosomes analyzed for each cell line per experiment. *P*-values are shown and generated from one-way ANOVA analysis followed by Tukey's multiple comparison.

*CAG-Cre^ER^; p53^F/F^; Pot1a^F/F^; Pot1b^+/−^* mice were generated from multiple-step cross-mating between *Pot1a^F/F^* mice (10), *Pot1b^−/−^* (22), *p53^F/F^* mice and *CAG-Cre^ER^* mice (Jackson Laboratory). Primary Mouse Embryonic Fibroblasts (MEFs) *CAG-Cre^ER^; p53^F/F^; mPot1a^F/F^; mPot1b^+/−^* and *CAG-Cre; p53^F/F^; mPot1a^F/F^; mPot1b^−/−^* were isolated from embryos generated from cross-mating between *CAG-Cre^ER^; p53^F/F^; Pot1a^F/F^; Pot1b^+/−^ and p53^F/F^; Pot1a^F/F^; Pot1b^+/−^* mice. After treatment with 4-hydroxy-Tamoxyfen (4-HT) or Adeno-Cre and passaging for 3 months, *p53* was completely deleted and *Pot1a* was partially removed in immortalized MEFs. Primary *Pot1b^−/−^* MEFs were immortalized with SV40 to generate the *Pot1b^−/−^; p53^−/−^* MEF cell line. The injection procedure is described in detail in Figure 1A. G1 sarcoma cell lines were harvested from ICR-SCID mice sarcoma by subcutaneous injections of G0 MEFs into 8-week-old ICR-SCID mice. Subsequent generations of sarcoma cell lines were isolated from the repeated injections of sarcoma cells into ICR-SCID mice. All mice were maintained according to the IACUC-approved protocols of Yale University. All mouse cell lines were cultured in DMEM/high glucose media supplemented with 7% FBS. The 293T cell line used to generate virus was cultured in DMEM/high glucose media supplemented with 7% FBS. The U2OS cell line was cultured in McCoy's 5A media supplemented with 7% FBS.

### Plasmids, retrovirus and antibodies

POT1a, POT1b and hPOT1 mutants were generated by PCR and constructed in the retrovirus expression vector pQCXIP-puro (23). Mouse telomerase expression vector HA-mTerT/ pMGIB was a gift from Steven Artandi and human telomerase RNA expression vector pBABEpuro-U3-hTR-500 was purchased from Addgene (#27666) (40). TERT K78E and K560N mutants were generated using an Agilent Site-directed mutagenesis kit. Fucci-mKO-CDT-1 and Fucci-mAG-Geminin plasmids were a gift from Atsushi Miyawaki (41). pBabe puro mTRF2^ΔBΔM^ and pQXCIP puro mTPP1^ΔRD^ (either MYC- or HA-tagged) were used to remove endogenous TRF2 and POT1-TPP1, respectively (42). The phCMV1_2xMBP_MCS plasmid used for POT1 purification was a gift from Ryan Jensen (43). All lentivirus and retrovirus were generated in 293T cells and used to infect target cells twice. Primary antibodies used for immunofluorescence were used in 1:1000 dilutions and include: anti-phospho-γH2AX (S139) (Millipore, #05–636), anti-pRPA32 (S33) (Bethyl, #A300-246A), PML (Santa Cruz, H-238, #sc-5621), anti‐STN1 (generated in Chang laboratory (17)) and anti-mTRF2 (gift from Jan Karlseder, 1:1000 dilution). Anti-epitope tag antibodies were purchased from Sigma (anti-Flag #F3165 and anti-HA #A300-305A) or Millipore (anti-Myc #05-724). Secondary antibodies for immunostaining were purchased from Invitrogen and used at a 1:2000 dilution: Alexa Fluor 350 anti-rabbit (A11044), Alexa Fluor 350 anti-mouse (A11045), Alexa Fluor 488 anti-mouse (A11001), Alexa Fluor 568 anti-mouse (A11004), Alexa Fluor 488 anti-rabbit (A11008), Alexa Fluor 594 anti-rabbit (A11012). Anti-γ-tubulin antibody (GTU-88, Sigma, #T6557, 1:5000 dilution) was used for the internal control in western blots. Secondary antibodies for western blots: peroxidase-linked anti-mouse IgG (Amersham NXA931V, 1:5000 dilution), peroxidase-linked anti-rabbit IgG (Amersham NA934V, 1:5000 dilution). Aphidicolin (#A0781). ATR inhibitor AZ20 (BAY1895344, gift from Ranjit Bindra).

### Disruption of *mTert* with CRISPR/Cas9

We utilized the CRISPR/Cas9 constructs described in Gu *et al.* (23). Mouse telomerase guide RNA mTert sgRNA [5′-GCTACGGGAGCTGTCAC(PAM)-3′] was designed online (https://chopchop.rc.fas.harvard.edu/) and cloned into lentiviral Cas9 vector LentiCRISPRv2puro (Addgene #89290) and delivered into G7 *p53^−/−^; Pot1a^F/−^; Pot1b^−/−^* sarcoma cells by infection of lentivirus produced in 293T cells. Infected cells were selected by puromycin for several days and single clones were picked and amplified. The targeted clones were screened by limited dilution and disruption of the gene was confirmed by sequencing of Topo TA-cloned PCR products of the targeting locus [PCR primers:5′- CTGCATGCTCCTGTCATAACTC-3′ and 5′- GACTCAACCATCAGTACAGGGG-3′].

### Telomere signal analysis by telomere PNA-FISH and CO-FISH

Cells were treated with 0.5 μg/ml of Colcemid (Invitrogen) for 4 h before harvest. Chromosomes were fixed with 3% formamide and hybridized with a telomere PNA-FISH 5′-Cy3-OO-(CCCTAA)_3_–3′ probe (PNAgene) as described in Wu *et al.* (10). DNA was counterstained with DAPI. Digital images were captured using NIS-Elements BR (Nikon) with a Nikon Eclipse 80i microscope utilizing an Andor CCD camera. The relative telomere signals were analyzed with ImageJ software (downloaded from Fiji). For CO-FISH, cells were incubated with 10 μM BrdU for 12 h, treated with 0.5 μg/ml of Colcemid for 4 h and harvested. Formalin-fixed metaphase spreads were stained with 0.5 μg/ml of Hoechst 33258 (Sigma) in 2 × SSC for 15 min at room temperature before being exposed to UV light equivalent to 5.4 × 10^3^ J/m^2^. After digestion with 200 U of Exonuclease III (Promega), the samples were denatured at 85°C for 3 min and incubated sequentially with 5′-Cy3-OO-(CCCTAA)_3_–3′ and 5′-FAM-OO-(TTAGGG)_3_–3′ probes as described above. Images were captured as described above.

### Immunofluorescence and fluorescent *in situ* hybridization

Cells were attached to slides by cytospin, fixed for 10 min in 2% (w/v) sucrose and 2% (v/v) paraformaldehyde at room temperature followed by PBS washes and permeabilized with 0.5% NP40 for 10 min followed by 0.5% TritonX for 5 min. Slides were blocked overnight in blocking solution (0.2% (w/v) fish gelatin and 0.5% (w/v) BSA in 1x PBS at 4°C. The cells were incubated with primary antibodies overnight at 4°C. After 0.1% Triton-PBS washes, slides were incubated with the appropriate Alexa fluor secondary antibody for 1 h followed by washes in 1× PBS with 0.1% Triton. PNA-FISH was carried out as above using a PNA telomere probe 5′-Cy3-OO-(CCCTAA)_3_-3′ (PNAgene). Digital images were captured as described above.

### Cell-cycle-dependent TIF analysis

G7 *Pot1b^−/−^* sarcoma cells were infected with Fucci-CDT1 or Fucci-geminin lentivirus and maintained for 3 days. Then the cells were processed for immunostaining with γ-H2AX and mTRF2 antibodies. The cells were mounted under coverslips without DAPI mounting reagent before taking images.

### Detection of telomerase RNA (TR) by RNA-FISH in mouse cells

G3 *Pot1b^−/−^* and G7 *Pot1b^−/−^* sarcoma cells were co-infected with retroviruses expressing mouse telomerase from vector HA-mTert/pMGIB and human telomerase RNA from vector pBABEpuro-U3-hTR-500. After double selection with 10 μg/ml of blasticidin and 2 μg/ml of puromycin for several days, the super-mTert/hTR cells were established and used for telomerase recruitment assays. Following immunostaining with antibody, cells were incubated in prehybridization solution (0.1% Dextran sulfate, 1 mg/ml of BSA, 2x SSC, 50% formamide, 0.5 mg/ml spermidine DNA, 0.1 mg/ml *Escherichia coli* tRNA, 1 mM RNase inhibitor VRC) at 37°C for 1 h_,_ then hybridized with combined PNA-FISH Cy3-OO-(CCCTAA)_3_ and Cy5-hTR cDNA probes in prehybridization solution overnight at 37°C. The washing conditions are the same as described for PNA-FISH. Digital images were captured as above.

### TERRA FISH

Cells grown on coverslips were treated with cytobuffer (100 mM NaCl, 300 mM sucrose, 3 mM MgCl_2_, 10 mM PIPES pH7.0, 0.1% Triton X-100, 10 mM vanadyl ribonucleoside complex Sigma #R3380) for 7 min at 4°C. Cells were rinsed briefly, fixed with 4% paraformaldehyde in PBS for 10 min at RT. Cells were then washed three times with PBS for 5 min each and then incubated with hybridization mix (10 nM TERRA FISH probe 5′-(TAACCC)_7_-Alexa488-3′, Integrated DNA Technologies, 50% formamide, 2× SSC, 2 mg/ml BSA, 10% dextran sulfate, 10 mM vanadyl ribonucleoside complex) for 18 h in a humidified chamber at 39°C. Cells were washed with 2× SSC in 50% formamide three times at 39°C for 5 min each, three times in 2 × SSC at 39°C for 5 min each, and finally once in 2× SSC at RT for 10 min. Coverslips were than mounted on glass microscope slides with DAPI. Digital images were captured as described above.

### TRF Southern analyses

A total of 2 × 10^6^ cells were suspended in PBS, 1:1 mixed with 1.8% agarose in 1x PBS and cast into plugs. The plugs were digested at 55°C for 2 days with 1 mg/ml proteinase K (Roche) in 10 mM sodium phosphate (pH7.2) and 0.5 mM EDTA and 1% sodium lauryl sarcosine. After completely washing away proteinase K with TE buffer, the DNA in plugs were subsequently digested with RsaI and HinfI overnight at 37°C. Plugs were washed 3× at 55°C in ExoI buffer (67 mM Tris–HCl, 6.7 mM MgCl_2_, 10 mM β-mercaptoethanol, 0.1 mg/ml Bovine Serum Albumin) with 150 mM of either KCl or LiCl. Plugs were cooled and then digested overnight at 37°C in ExoI buffer. Plugs were loaded onto a 0.8% pulse-field agarose (Bio-Rad) gel in 0.5x TBE and electrophoresed on a CHEF-DRII pulse field electrophoresis apparatus (Bio-Rad). The electrophoresis conditions were as follows: initial pulse 0.3 s, final pulse 16 s, voltage 6 V/cm, run time 14.5 h. The gels were dried and pre-hybridized in Church mix (0.5 M NaH_2_PO_4_, pH7.2, 7% SDS), and then hybridized with telomeric repeat oligonucleotide probes γ-^32^P-(CCCTAA)_4_ or γ-^32^P-(TTAGGG)_4_ in Church mix at 55°C overnight. Gels were washed with 4× SSC, 0.1% SDS buffer at 55°C and exposed to Phosphorimager screens overnight. The screen was scanned on a Typhoon Trio image system (GE Healthcare). For quantification of total telomeric DNA, the gels were de-probed with denaturing solution 0.5 M NaOH, 1.5 M NaCl and neutralized with 3 M NaCl, 0.5 M Tris–Cl, pH7.0, and re-probed with telomeric γ-^32^P-(CCCTAA)_4_ or γ-^32^P-(TTAGGG)_4_ probes. Quantification of signal intensity was performed on ImageJ. Native signals were normalized to total telomeric signal.

### 2D-TRF Southern assay

TRF Southern plugs were prepared as described above and digested with Hinf1/Rsa1 before resolving them in the first dimension in 0.4% agarose gel in 0.5× TBE at 26 V for 15 h. Ethidium bromide-stained gel slices were rotated 90° from the first gel and cast in a 1% agarose gel and resolved at 115 V for 4 h. Dried gels were denatured and subsequently hybridized as described above. After exposure, hybridization signals were analyzed with a Typhoon Trio imager system and ImageQuant TL software.

### TRAP assay

TRAP assays were performed as described in the manufacturer's protocol (Millipore #S7700). Briefly, 1 × 10^6^ cells were lysed with 200 μl of CHAPS buffer and the soluble fractions were freshly frozen in liquid nitrogen and stored at −80°C. The telomerase reaction was carried out in 25 μl TRAP buffer containing: 20 mM Tris–Cl (pH8.0), 63 mM KCl, 1.5 mM MgCl_2_, 1 mM EGTA, 2 ng/μl of γ-^32^P labeled TS primer (AATCCGTCGAGCAGAGTT), 50 μM 4dNTP and 1x TRAP primer mix at 30°C for 40 min and quenched at 95°C for 2 min. Immediately following the addition of Taq DNA polymerase, the PCR was performed as follows: 94°C 30 s, 59°C 30 s repeating for 28 times. The PCR reaction was separated on 10% acrylamide gel in 0.5× TBE (Invitrogen EC62752) and electrophoresed for 81 min under 150 V. The gel was dried and exposed to phosphorimager screen. The radioactive signals were captured and quantified as described for TRF Southern blotting.

### C-circle assay

The C-circle assay was performed as described in Henson *et al.* (44). Briefly, genomic DNA was digested by RsaI and HinfI overnight at 37°C. The digested DNA was precipitated using ethanol and resuspended in H_2_O. Digested DNA was then amplified using Phi29 polymerase (New England Biolabs M0269S). The amplified product was then immobilized to an Amersham Hybond N^+^ membrane using a slot dot blot apparatus. C-circle products were then detected by hybridization with γ-^32^P-(CCCTAA)_4_ telomeric probes.

### Western blot analyses

Trypsinized cells were lysed in urea lysis buffer (8 M urea, 50 mM Tris–HCl, pH 7.4, and 150 mM β-mercaptoethanol). The lysates were denatured and then resolved on an SDS-PAGE gel. The separated proteins were then blotted on a nitrocellulose plus membrane (Amersham), blocked with blocking solution (5% non-fat dry milk in PBS/0.1% Tween-20) for at least 1 h and incubated with the appropriate primary antibody in blocking solution at least 2 h at room temperature or overnight at 4°C. The membranes were washed 3 × 5 min with PBS/0.1% Tween-20 and incubated with appropriate secondary antibody in blocking solution for 1 h at room temperature. Chemiluminescence detection was performed using an ECL Western Blotting Detection kit from GE Healthcare.

### POT1 protein purification

Human POT1 and mouse POT1a or POT1b cDNAs were subcloned into a phCMV1_2xMBP_MCS vector (Jensen *et al.*, 2010) (43). The phCMV1 construct with 2xMBP-tagged hPOT1 and vector containing 1x Flag-tagged hTPP1 were transiently co-transfected using LipoD293 into 293T cells. The phCMV1 construct with 2xMBP-tagged mPOT1a or mPOT1b and vector with HA-tagged mTPP1 were transiently co-transfected into 293T cells. All the purification steps were carried out at 4°C using a protocol as described (Jensen, 2014) (45) with modifications. In brief, the cells were harvested 32 h post-transfection and lysed in buffer A (50 mM HEPES (pH 7.5), 150 mM NaCl, 5 mM EDTA, 1% Igepal CA-630, protease inhibitor cocktail (Roche), 1 mM PSMF and 1 mM DTT). The lysate was cleared by ultracentrifugation for 20 min at 10000 g, and the supernatant was batch bound to amylose resin (New England Biolabs) overnight. The POT1-TPP1 complex proteins were then eluted with buffer B (50 mM HEPES (pH 8.2), 150 mM NaCl, 0.5 mM EDTA, 10% glycerol, 1 mM DTT, protease inhibitors, 0.01% Igepal CA-630 and 1 mM PSMF) with 50 mM maltose. For human POT1-TPP1 complex, the eluate was mixed with anti-FLAG M2 Affinity Agarose Gel (Sigma) for 2hrs, followed by protein elution in elution buffer B with 0.3 mg/ml 1xFlag peptide (Sigma). The eluates were concentrated to 500 ng/ul in a 2 ml 100 kDA Centrifugal Filter (Amicon) and stored in 2 μl aliquots at −80°C.

### Telomeric oligonucleotides and G-quadruplex preparation

The telomeric DNA for G-quadruplex formation was performed as described (46). The oligo sequences are: 5′-TTAGGGTTAGGGTTAGGG-3′ which cannot form G-quadruplexes and 5′-GGGTTAGGGTTAGGGTTAGGG-3′ which can form G-quadruplexes. The 5′ P^32^-radiolabeled DNA was annealed in buffer C (100 mM NaCl, 50 mM Tris–HCl [pH7.9], 10 mM MgCl_2_, 1 mM DTT) by heating to 95°C for 5 min and then snap-cooled on ice for 5 min.

### Telomeric DNA binding assay

POT1-TPP1 proteins (50–200 nM) were incubated with radiolabeled telomeric DNA (50nM, oligonucleotides) in 10 μl reaction buffer D (25 mM Tris–HCl [pH 7.5], 50 mM KCl, 1 mM DTT, 100 mg/ml BSA, and 1.5 mM MgCl_2_) at 37C for 10 min. The reaction mixtures were resolved in 10% polyacrylamide gels in 1× Tris–borate–EDTA (TBE) buffer (40 mM Tris–HCl [pH 8.3], 45 mM boric acid, and 1 mM EDTA) at 4°C. After gel-drying, the radiolabeled DNA species were visualized by phosphorimaging analysis.

### G-quadruplex unfolding assay

POT1-TPP1 proteins (50–200 nM) were incubated with radiolabeled G-quadruplex DNA (50 nM, of 5′-GGGTTAGGGTTAGGGTTAGGG-3′) in 10 μl reaction buffer E (20 mM HEPES [pH 7.5], 100 mM NaCl, 0.1 mM DTT, 100 mg/ml BSA and 2 mM MgCl_2_) at 37°C for 10 min. Then the reactions were incubated for 10 min at 37°C after adding SDS (0.2%) and proteinase K (0.25 mg/ml). The deproteinized reaction mixtures were separated on 10% polyacrylamide gels in 0.5× TBE buffer. A control oligonucleotide in LiCl buffer (150 mM LiCl, 67 mM Tris–HCl, 6.7 mM MgCl_2_, 10 mM β-mercaptoethanol, 0.1 mg/ml Bovine Serum Albumin) without G-quadruplex formation was used as an unfolded DNA control. Gels were dried and subjected to phosphorimaging analysis.

### Statistical analyses

Statistical analyses were carried out using the Prism software. Asterisks represent the degree of significance determined by the stated statistical test: *, **, *** and **** denote < 0.05, <0.01, <0.001 and < 0.0001 *P*-values, respectively. Error bars indicate the standard deviation unless otherwise noted.

## RESULTS

### Telomere hyper-elongation in late-generation *Pot1b^−/−^* tumors

We previously generated *Pot1a^F/Δ^; Pot1b^+/−^; p53^Δ/Δ^* (abbreviated *Pot1b^+/−^*) and *Pot1a^F/Δ^; Pot1b^−/−^; p53^Δ/Δ^* (abbreviated *Pot1b^−/−^*) mouse embryonic fibroblasts (MEFs) (23). *Pot1b^+/−^* and *Pot1b^−/−^* MEFs formed sarcomas after subcutaneous injection into flanks of severe combined immunodeficient (SCID) mice. These sarcomas were harvested to generate first-generation (G1) sarcoma cell lines. Following serial transplantation of 100 000 cells into SCID mice, we established three subsequent generations of *Pot1b^−/−^* and *Pot1b^+/−^* cell lines (23). Using quantitative telomere-FISH (Q-FISH), we discovered that telomere lengths progressively shorten in each subsequent sarcoma generation of *Pot1b^−/−^* but not *Pot1b^+/−^* cells (Supplementary Figure S1a–c), in accord with previous results (21,22). To examine whether *Pot1b^−/−^* sarcomas are ultimately able to overcome this telomere shortening phenotype by activating a telomere length maintenance pathway, we continued serial transplantation of *Pot1b^+/−^* and *Pot1b^−/−^* sarcomas into SCID mice for up to seven generations (G7) (Figure 1A, Supplementary Figure S1a). Surprisingly, G4 *Pot1b^−/−^* cells displayed progressively elongated telomeres, with an over 1.9-fold increase in length compared to G3 *Pot1b^−/−^* cells as determined by both telomere restriction fragment (TRF) Southern and Q-FISH analyses (Figure 1B–D). Telomere elongation continued with each subsequent generation of serially transplanted sarcomas, culminating with telomere hyper-elongation observed in G7 *Pot1b^−/−^* cells where the mean telomere FISH fluorescent intensity measured 1.4-fold longer than those observed in G7 *Pot1b^+/−^* cells. To confirm this surprising phenotype, we generated an independent *Pot1b^−/−^; p53^−/−^* MEF cell line and discovered that it also underwent telomere hyper-elongation following twelve generations of serial transplantation (Supplementary Figure S1d, e). In contrast, telomeres in late generation *Pot1b^+/−^* sarcomas never elongated (Figure 1B–D). These observations suggest that long-term passaging of sarcomas in the absence of *Pot1b* disrupts telomere length homeostasis to promote telomere hyper-elongation.

In contrast to *Pot1b^+/−^* sarcomas, G1-G3 *Pot1b^−/−^* sarcomas display chromosomal fusions, with up to 14% of chromosomes containing Robertsonian chromosomal fusions without telomeric signals at the fusion site (Figure 1C, E). These types of end-to-end chromosomal fusions are indicative of the critical telomere shortening observed in the absence of *Pot1b* (21,22,47). However, the number of chromosomal fusions did not increase in G4 to G7 *Pot1b^−/−^* sarcomas, remaining at ∼14% of all chromosomes (Figure 1C, E). We speculate that telomere hyper-elongation acquired in later generations is counteracting the initial telomere shortening to prevent further accumulation of fused chromosomes. The Robertsonian translocations accrued during G1–G3 would be stable and heritable as they contain only one centromere and so the chromosomal fusions would be retained in late-generation *Pot1b^−/−^* cells. To evaluate the ability of hyper-elongated telomeres from G7 *Pot1b^−/−^* cells to inhibit DNA repair, we expressed the dominant negative shelterin proteins TPP1^ΔRD^ and TRF2^ΔBΔM^ (12,48). TPP1^ΔRD^ and TRF2^ΔBΔM^ expression had no effect on the number of chromosomal fusions without telomeric signals but led to a 15% increase of chromosomal fusions with telomeric signals, suggesting that expressing these two proteins deprotected telomeres in both G7 *Pot1b^+/−^* and *Pot1b^−/−^* cells (Supplementary Figure S2a, d–f). These results suggest that hyper-elongated telomeres in G7 *Pot1b^−/−^* cells repress aberrant chromosomal fusions similar to those in *Pot1b^+/−^* cells. We next set out to determine whether hyper-elongated telomeres are able to protect against activation of a DDR. G7 *Pot1b^−/−^* cells had detectable γ-H2AX-positive telomere dysfunction induced foci (TIFs) in ∼22% of nuclei (Supplementary Figure S2a–c). Altogether, these results suggest that hyper-elongated telomeres in *Pot1b* null sarcomas remain protective against DNA repair at telomeres and prevent chromosomal fusions but are unable to fully repress DDR activation.

### Telomere hyper-elongation in late-generation *Pot1b^−/−^* tumors is mediated by telomerase

**Figure 2.**
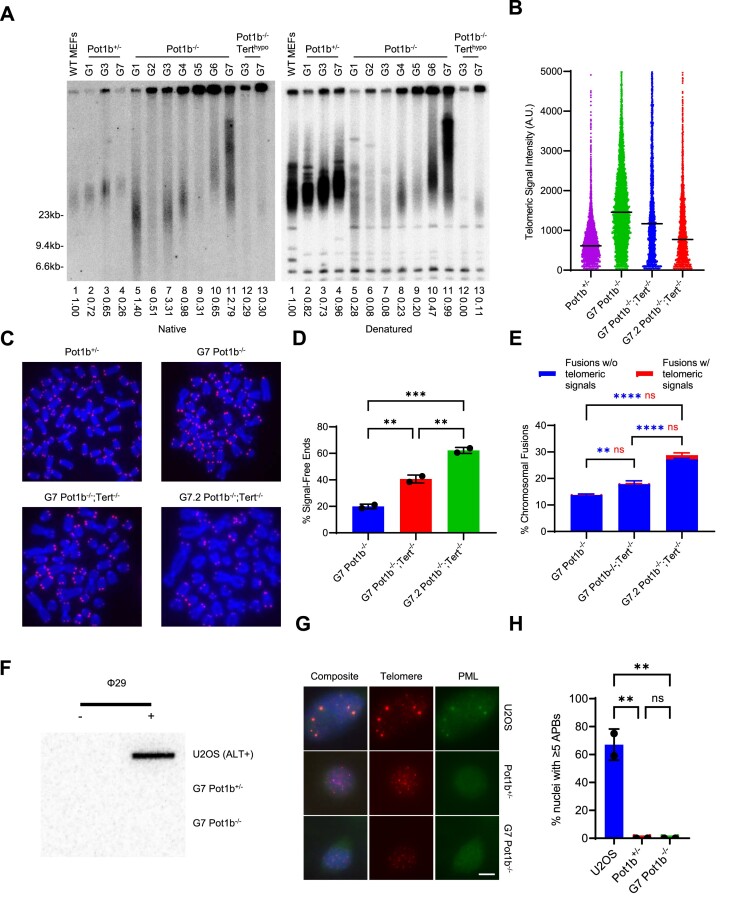
Telomere hyper-elongation is mediated by telomerase. (**A**) TRF Southern blot detection of G-overhang in native gel and total telomere length in denatured gel hybridized with γ-^32^P-(CCCTAA)_4_ telomere probe. Numbers indicate relative G-overhang and total telomere signals, with telomere signals set to 1.0 for WT MEFs. Molecular weight markers as indicated. (**B**) Q-FISH analysis showing the median telomeric signal intensity from metaphases in (C). 30 metaphases were scored per cell line. (**C**) Representative images of metaphase spreads of the indicated cell lines visualized with PNA-FISH probe Cy3-OO-(CCCTAA)_3_ and DAPI. Scale bar: 5 μm. (**D**) Quantification of signal-free ends in metaphases in (C). Data show the mean ± standard deviation from two independent experiments with 30 metaphases analyzed for each cell line per experiment. *P*-values are shown and generated from one-way ANOVA analysis followed by Tukey's multiple comparison. (**E**) Quantification of chromosomal fusions from metaphases in (C). Data show the mean ± standard deviation from two independent experiments with 30 metaphases analyzed for each cell line per experiment. *P*-values are shown and generated from two-way ANOVA analysis followed by Tukey's multiple comparison. Blue *P*-values are used for chromosomal fusions without telomeric signals. Red *P*-values are used for chromosomal fusions with telomeric signals. (**F**) C-circle detection in the indicated cell lines. Slot-blot of C-circle assay performed on genomic DNA with and without Phi29 DNA polymerase. U2OS was used as a positive control. (**G**) Representative images of APBs in the indicated cell lines. Cells were immunostained with α-PML antibody (green), telomere probe Cy3-(CCCTAA)_3_ (red) and DAPI to stain nuclei (blue). Scale bar: 5 μm. (**H**) Quantification of the indicated cell lines in (G) with α-PML foci colocalizing with telomeres. Data show the mean ± standard deviation from two independent experiments with 150 nuclei analyzed for each cell line per experiment. *P*-values are shown and generated from one-way ANOVA analysis followed by Tukey's multiple comparison. U2OS was used as a positive control.

Telomere hyper-elongation can be mediated either by telomerase or via the Alternative Lengthening of Telomeres (ALT) pathway (49). To determine if telomerase is responsible for the elongating telomeres in late-generation *Pot1b^−/−^* cells, we utilized a G3 *Pot1b^−/−^* cell line with hypomorphic telomerase activity (*Pot1b^−/−^; Tert^hypo^*) that we had previously generated using CRISPR/Cas9 (23). TRF Southern analysis revealed that serial transplantation of G7 *Pot1b^−/−^; Tert^hypo^* cells failed to induce telomere hyper-elongation (Figure 2A). These results suggest that WT level of telomerase activity is required for *Pot1b^−/−^* cells to undergo telomere hyper-elongation. To further confirm a role for telomerase in telomere hyper-elongation, we used CRISPR/Cas9 to knockout *Tert* in G7 *Pot1b^−/−^* cells (G7 *Pot1b^−/−^*; *Tert^−/−^*) (Supplementary Figure S3a–c). Telomere Q-FISH analysis following serial transplantation of G7 *Pot1b^−/−^; Tert^−/−^* cells revealed that deletion of telomerase leads to rapid telomere erosion, with the average telomere intensity decreasing by 36% (Figure 2B, C). In addition, both telomere-free chromosome ends and chromosome fusions without telomeric signals increased by 1.5-fold, further revealing the necessity of telomerase in late-generation *Pot1b^−/−^* cells for telomere hyper-elongation (Figure 2C–E).

Two phenotypes associated with high specificity for ALT are extrachromosomal telomeric C-circles and ALT-associated PML nuclear bodies (APBs) (44,50). Late-generation *Pot1b^−/−^* cells displayed no detectable C-circles or APBs (Figure 2F–H). In addition, 2D-gel analysis of late-generation *Pot1b^−/−^* cells had no detectable extrachromosomal telomeric DNA such as telomere (T)-circles or recombination intermediates such as telomere (T)-complexes (Supplementary Figure S3d, e) (51). We also did not detect any increase in the rate of telomere sister chromatid exchange (T-SCE) another prominent phenotype observed in ALT cells (Supplementary Figure S4a, b) (52–54). Furthermore, there was no evidence at telomeres in G7 *Pot1b^−/−^* cells of increased replication stress or telomeric repeat-containing RNA (TERRA) transcription, phenotypes thought to drive ALT activity (Supplementary Figure S4c–e) (55–57). The absence of ALT phenotypes strongly suggests that the telomere hyper-elongation of late-generation *Pot1b^−/−^* cells is mediated by telomerase rather than through the activation of ALT.

### Telomerase recruitment to telomeres is increased in late-generation *Pot1b^−/−^* cells

**Figure 3.**
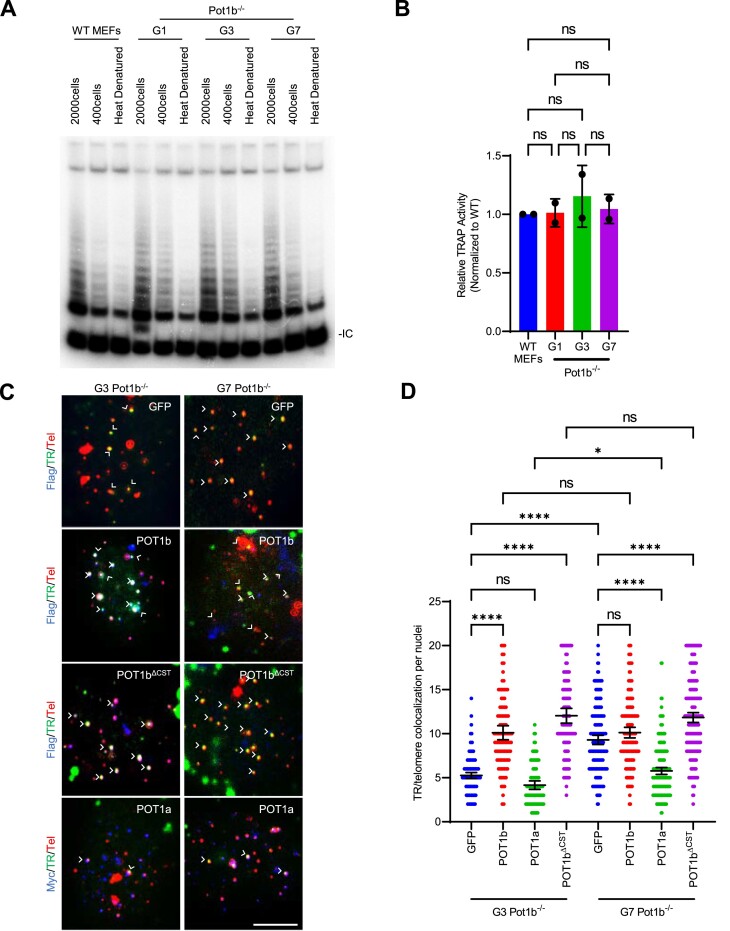
Increased telomerase recruitment to telomeres in late-generation *Pot1b^−/−^* cells. (**A**) TRAP assay of the indicated cell lines. WT MEFs were used as a positive control and heat denatured lysates as negative controls. IC: Internal Control. (**B**) Quantification of relative TRAP activity in (A). Values are normalized to WT levels and show the mean ± standard deviation. p-values are shown and generated from one-way ANOVA analysis followed by Tukey's multiple comparison. (**C**) Representative images of telomerase recruitment assay. Flag-POT1b or Myc-POT1a (blue) were detected by immunostaining with anti-Flag or anti-Myc antibodies. hTR RNA was detected by hybridization with Cy5-hTR cDNA probes (green) and telomeres visualized by hybridization with PNA probe Cy3-OO-(CCCTAA)_3_ (red). White arrows: TR/Telomere foci colocalization. Scale bar: 5 μm. (**D**) Quantification of telomerase and telomere colocalization in (C). Data show the mean ± 95% CI of the mean of one experiment with 150 nuclei analyzed per cell line.

We next set out to identify the mechanism underlying telomerase-mediated telomere hyper-elongation. We first examined whether late-generation *Pot1b^−/−^* cells have increased telomerase transcription. *TERT* promoter mutations are known to increase telomerase transcription in many cancers (58–62). However, sequencing the *Tert* promoters in G1 and G7 *Pot1b^−/−^* cells did not reveal any mutations (data not shown). To evaluate if telomerase enzymatic activity is increased in G7 *Pot1b^−/−^* sarcomas, we performed a quantitative Telomere Repeat Amplification Protocol (TRAP) assay (7). Compared to G1-G3 *Pot1b^−/−^* sarcomas, telomerase enzymatic activity did not increase appreciably in G7 *Pot1b*^*−/−*^ cells (Figure 3A, B). We next examined whether telomerase recruitment to telomeres was increased in G7 *Pot1b^−/−^* sarcomas (40). Compared to G3 *Pot1b^−/−^* cells, we detected a 1.8-fold increase in telomerase co-localization with telomeres in G7 *Pot1b^−/−^* cells (Figure 3C, D), suggesting that the increased telomerase recruitment contributed to telomere hyper-elongation. Since telomerase recruitment is mediated through its interaction with TPP1’s TEL patch and NOB domains (63–65), we sequenced these domains for mutations that might promote telomerase recruitment but did not detect any (data not shown).

We have recently shown that POT1b plays an important role in telomerase recruitment to telomeres (23) and speculated that overexpression of POT1b might give us some insight into the changes in telomerase recruitment dynamics in G7 *Pot1b^−/−^* cells. We overexpressed POT1b^WT^, POT1b^ΔCST^ (a mutant which cannot recruit the CST complex to telomeres) and POT1a^WT^ in G3 *Pot1b^−/−^* and G7 *Pot1b^−/−^* cells and measured telomerase recruitment. Interestingly, both G3 and G7 *Pot1b^−/−^* cells exhibited similar rates of telomerase recruitment, with approximately 10.1 TR/telomere co-localizations per nucleus upon overexpression of POT1b^WT^, as well as ∼12 co-localizations per nucleus when overexpressing POT1b^ΔCST^ (Figure 3C, D). Since G7 *Pot1b^−/−^* cells no longer have increased telomerase recruitment compared to G3 *Pot1b^−/−^* cells following overexpression of POT1b constructs, it is likely that POT1b represses the pathway leading to increased telomerase recruitment between late- and early-generations. In contrast, expressing POT1a^WT^ decreased telomerase recruitment in both G7 and G3 *Pot1b^−/−^* cells but remained elevated in G7 compared to G3 with 5.8 to 4.2 co-localizations per nucleus respectively (Figure 3C, D).

### Elevated DNA damage in G7 *Pot1b^−/−^* telomeres

**Figure 4.**
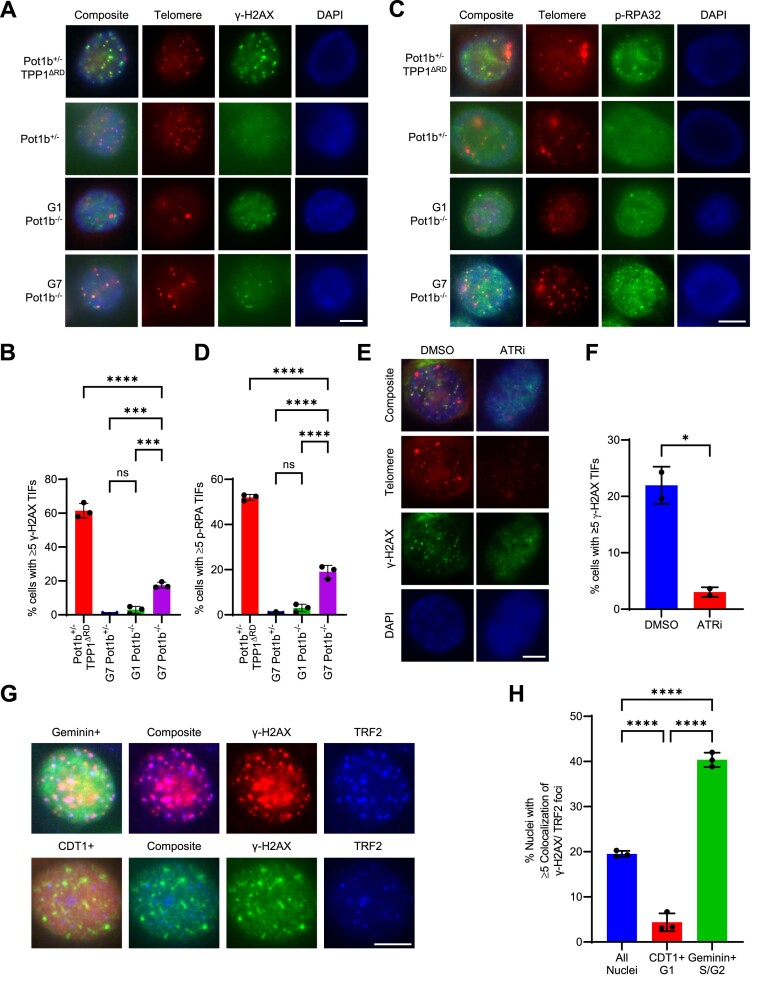
Elevated DNA damage detected in late-generation *Pot1b^−/−^* cells. (**A**) Representative image of γ-H2AX TIFs in the indicated cell lines. Cells were immunostained with γ-H2AX antibody (green), telomere probe Cy3-(CCCTAA)_3_ (red) and DAPI to stain nuclei (blue). Scale bar: 5 μm. (**B**) Quantification of the indicated cell lines with ≥5 γ-H2AX TIFs in (A). Data show the mean ± standard deviation from three independent experiments in which 150 nuclei were analyzed per cell line. *P*-values are shown and generated from one-way ANOVA analysis followed by Tukey's multiple comparison. (**C**) Representative images of p-RPA TIFs in the indicated cell lines. Cells were immunostained with p-RPA antibody (green), telomere probe Cy3-(CCCTAA)_3_ (red) and DAPI to stain nuclei (blue). Scale bar: 5 μm. (**D**) Quantification of the indicated cell lines with ≥5 p-RPA TIFs in (C). Data show the mean ± standard deviation from three independent experiments in which 150 nuclei were analyzed per cell line. p-values are shown and generated from one-way ANOVA analysis followed by Tukey's multiple comparison. (**E**) Representative images of γ-H2AX TIFs in G7 *Pot1b^−/−^* cells treated with DMSO or AZ20 150 nM for 24hr. Cells were immunostained with γ-H2AX antibody (green), telomere probe Cy3-(CCCTAA)_3_ (red) and DAPI to stain nuclei (blue). Scale bar: 5 μm. (**F**) Quantification of the indicated cell lines with ≥5 γ-H2AX TIFs in (E). Data show the mean ± standard deviation from two independent experiments in which 150 nuclei were analyzed per cell line. *P*-values are shown and generated from an unpaired *t*-test. (**G**) Representative images of G7 *Pot1b^−/−^* cells expressing cell-cycle sensors Geminin or CDT1 and immunostained with γ-H2AX antibody and TRF2 antibody. Scale bar: 5 μm. (**H**) Quantification of G7 *Pot1b*^*−/−*^ cells expressing the indicated construct in (G) with ≥5 γ-H2AX TIFs. Data show the mean ± standard deviation from three independent experiments in which 150 nuclei were analyzed per cell line. *P*-values are shown and generated from one-way ANOVA analysis followed by Tukey's multiple comparison.

Elevated DDR at telomeres promotes telomerase recruitment to telomeres in diverse organisms (39,66–72). Deletion of *Pot1b* has not been shown to activate a robust DDR at telomeres (9,73). Consistent with these findings, G1 *Pot1b^−/−^* cells display no detectable γ-H2AX TIFs (Figure 4A, B). Measuring across *Pot1b^−/−^* sarcoma generations, γ-H2AX TIF formation was undetectable in G1 to G3 *Pot1b^−/−^* nuclei but ∼10% of G4 *Pot1b^−/−^* nuclei exhibited γ-H2AX TIFs with increasing TIF rates measured with each subsequent generation (Supplementary Figure S5a). In marked contrast to early-generations, G7 *Pot1b^−/−^* cells possess robust γ-H2AX TIFs detected in ∼20% of nuclei (Figure 4A, B). Furthermore, γ-H2AX TIFs were also detected in ∼9% of nuclei from a second independent late-generation *Pot1b^−/−^; Pot1a^+/+^* sarcoma cell line (Supplementary Figure S5b, c). The presence of TIFs in late-generation *Pot1b*^*−/−*^ sarcomas suggest that the hyper-elongated telomeres are in a state that is unable to repress DDR activation. *Pot1b* deletion has been shown to result in 3′ G-overhang extension (9,73), and we hypothesize that the extensive ss-telomeric overhang observed in G7 *Pot1b^−/−^* cells is being recognized as damaged DNA by the single-stranded binding complex replication protein A (RPA). In support of this notion, ∼17% of G7 *Pot1b^−/−^* nuclei, but not G1 *Pot1b^−/−^* nuclei, contain ≥ 5 p-RPA32 (S33) TIFs (Figure 4C, D). The presence of p-RPA32 (S33) TIFs suggests that the ss-telomeric DNA activates the ataxia-telangiectasia and Rad3 related (ATR) DDR pathway (74). To test this hypothesis, we treated late-generation *Pot1b^−/−^* cells with the ATR inhibitor AZ20 (ATRi). Treatment with ATRi decreased the number of γ-H2AX TIFs by 86%, indicating that the p-RPA/ATR pathway is robustly activated in G7 *Pot1b^−/−^* telomeres (Figure 4E, F).

Previous data reveal that there are different requirements for POT1-mediated telomere protection in the G1 and S/G2 phases of the cell cycle (13). We utilized the FUCCI system to determine whether the source of the DNA damage observed in G7 *Pot1b^−/−^* cells was limited to a particular stage of the cell cycle (41). γ-H2AX TIFs were detected in 40% of nuclei in S/G2, marked by Geminin expression, while only 4% of nuclei in the G1 phase, indicated by CDT1 expression, were TIF-positive (Figure 4G, H). These data indicate that the G7 *Pot1b^−/−^* telomeres are recognized as damaged ss-DNA in the S/G2 phase of the cell cycle and that hyper-elongated telomeres remain protective during G1. This suggests processing of the overhang during S/G2 in late-generation *Pot1b^−/−^* cells likely underlies this dysfunction.

### The N-terminus of POT1b represses a DDR in late-generation *Pot1b^−/−^* cells

**Figure 5.**
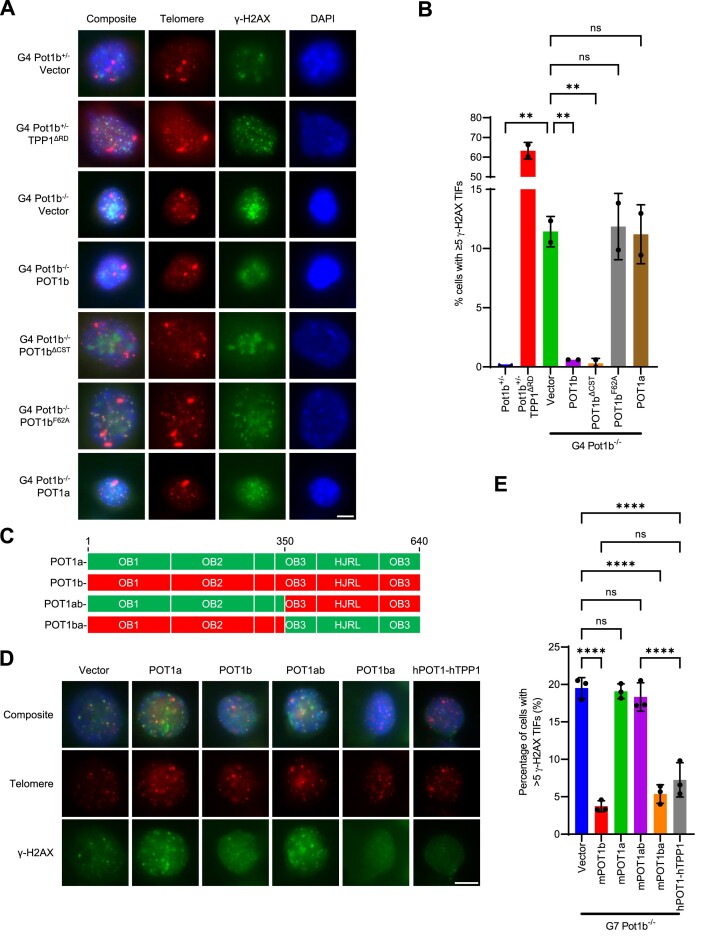
POT1b protects against telomeric DNA damage in late-generation *Pot1b^−/−^* cells. (**A**) Representative images of γ-H2AX TIFs in G4 *Pot1b*^*−/−*^cells expressing the indicated construct. Cells were immunostained with γ-H2AX antibody (green), telomere probe Cy3-(CCCTAA)_3_ (red) and DAPI to stain nuclei (blue). Scale bar: 5 μm. (**B**) Quantification of nuclei with ≥ 5 γ-H2AX TIFs from (A). Data show the mean ± standard deviation from two independent experiments in which 150 nuclei were analyzed per cell line. *P*-values are shown and generated from one-way ANOVA analysis followed by Tukey's multiple comparison. (**C**) Schematic of POT1a, POT1b, POT1ab and POT1ba chimera constructs. (**D**) Representative images of γ-H2AX TIFs in G7 *Pot1b^−/−^* cells expressing the indicated construct. Cells were immunostained with γ-H2AX antibody (green), telomere probe Cy3-(CCCTAA)_3_ (red) and DAPI to stain nuclei (blue). Scale bar: 5 μm. (**E**) Quantification of nuclei with ≥5 γ-H2AX TIFs from (D). Data show the mean ± standard deviation from three independent experiments in which 150 nuclei were analyzed per cell line. *P*-values are shown and generated from one-way ANOVA analysis followed by Tukey's multiple comparison.

POT1a and hPOT1 repress ATR activation at telomeres by binding to the G-strand ss-telomeric DNA to exclude RPA (13,14,75). Since p-RPA TIFs are present in G7 *Pot1b^−/−^* cells, we reasoned that endogenous POT1a cannot adequately protect the elongated ss-telomeric DNA. PCR genotyping revealed that *Pot1a* expression is intact in G7 *Pot1b^−/−^* cells (Supplemental Figure S1a). We next overexpressed POT1a or POT1b in the G4 *Pot1b^−/−^* cells to evaluate if increasing POT1 proteins could repress the DNA damage. Unexpectedly, overexpression of POT1b, but not POT1a, in G4 *Pot1b^−/−^* cells abolished both γ-H2AX and p-RPA TIF formation by ∼96% and ∼93%, respectively (Figure 5A, B, Supplementary Figure S5d–g). It has never been demonstrated previously that POT1b has a unique ability to repress a DDR that POT1a cannot. However, this result revealed that the DDR observed in G4 *Pot1b*^*−/−*^ cells are uniquely protected by POT1b but not by POT1a. We next set out to determine how POT1b is repressing TIFs. Unlike POT1a, POT1b is critical for the recruitment of the CST complex to telomeres to mediate C-strand synthesis (16). However, expression of POT1b^ΔCST^, a mutant unable to recruit the CST complex to telomeres (16), also repressed TIFs in late-generation *Pot1b^−/−^* cells (Figure 5A, B, Supplementary Figure S5d–g). We additionally confirmed that POT1b's DNA binding activity is required to repress TIFs through expression of POT1b^F62A^, an N-terminal mutant unable to bind to ss-telomeric DNA (10) (Figure 5A, B, Supplementary Figure S5d–g). To further dissect which domains of POT1b are required to repress TIFs, we overexpressed the POT1a^1–350^-POT1b^351–640^ chimeric protein (abbreviated POT1ab) or the POT1b^1–350^-POT1a^351–640^ protein (abbreviated POT1ba) (23) in G7 *Pot1b^−/−^* cells (Figure 5C). POT1ba but not POT1ab repressed γ-H2AX TIFs by ∼73%, confirming that the N-terminus but not the C-terminus of POT1b is required for POT1b's ability to repress γ-H2AX TIFs (Figure 5D, E, Supplementary Figure S6a, b). Importantly, the POT1ba chimera lacks critical amino acids required for recruitment of the CST-complex to telomeres (16) (Supplementary Figure S6c, d), further supporting the notion that POT1b's telomere protective function is independent of its role in CST recruitment. We found that overexpressing hPOT1 repressed γ-H2AX TIFs in G7 *Pot1b^−/−^* cells, suggesting that POT1b's protective function is conserved in hPOT1. We confirmed these results by overexpressing the chimeric POT1a/b and hPOT1 constructs in independently generated *Pot1b^−/−^; p53^−/−^* sarcomas, demonstrating that POT1ba and hPOT1 repress γ-H2AX TIFs to nearly undetectable levels (Supplementary Figure S6e–g). These results suggest that the DNA-protective ability of POT1b in G7 *Pot1b^−/−^* cells is distinct from that of POT1a.

### Telomere hyper-elongation in G7 *Pot1b^−/−^* cells is due to the activation of ATR-dependent DDR at telomeres

**Figure 6.**
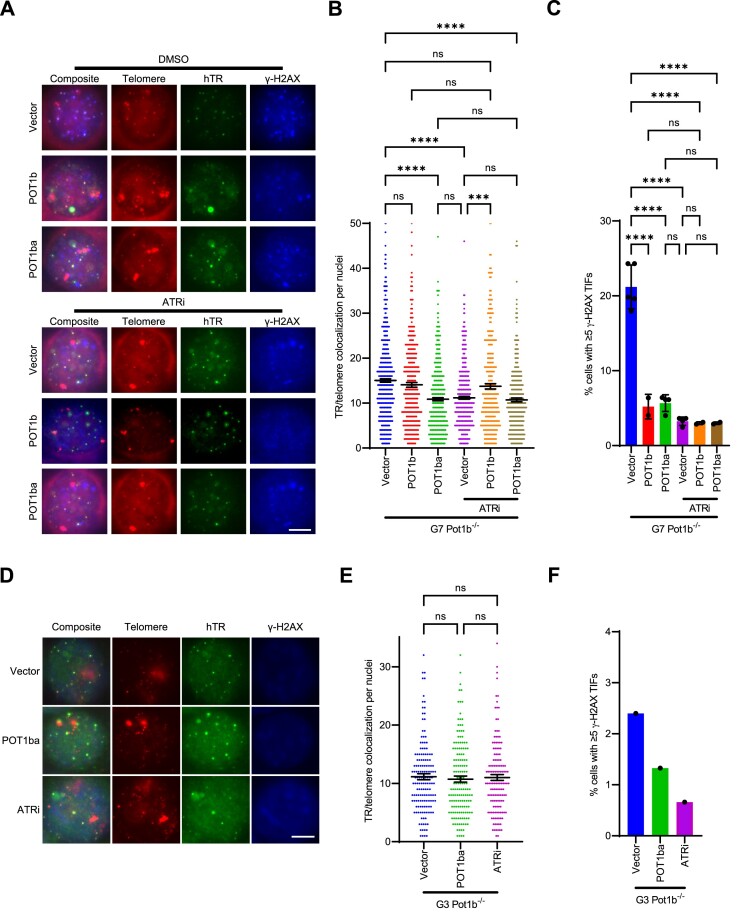
Increased telomeric damage in G7 *Pot1b*^*−/−*^ cells promotes telomerase recruitment to telomeres. (**A**) Representative images of telomerase recruitment assay in G7 *Pot1b*^*−/−*^ cells treated with/without 150nM AZ20, and with/without expressing vector/POT1b/POT1ba. γ-H2AX (blue) were detected by immunostaining with anti-γH2AX antibodies. hTR RNA was detected by hybridization with Cy5-hTR cDNA probes (green) and telomeres visualized by hybridization with PNA probe Cy3-OO-(CCCTAA)_3_ (red). Scale bar: 5μm. (**B**) Quantification of telomerase and telomere colocalization in (A). Data show the mean ± 95% CI of the mean from at least two experiments with 150 nuclei analyzed per cell line. p-values are shown and generated from one-way ANOVA analysis followed by Tukey's multiple comparison. (**C**) Quantification of the indicated conditions with ≥ 5 γ-H2AX TIFs in (A). Data show the mean ± standard deviation from at least two independent experiments in which 150 nuclei were analyzed per cell line. P-values are shown and generated from one-way ANOVA analysis followed by Tukey's multiple comparison. (**D**) Representative images of telomerase recruitment assay in G3 *Pot1b*^*−/−*^ cells treated with/without 150nM AZ20, with/without expressing POT1ba. γ-H2AX were detected by immunostaining with anti-γH2AX antibodies (blue). hTR RNA was detected by hybridization with Cy5-hTR cDNA probes (green) and telomeres visualized by hybridization with PNA probe Cy3-OO-(CCCTAA)_3_ (red). Scale bar: 5 μm. (**E**) Quantification of telomerase and telomere colocalization in (D). Data show the mean ± 95% CI of the mean with 150 nuclei analyzed per cell line. P-values are shown and generated from one-way ANOVA analysis followed by Tukey's multiple comparison. (**F**) Quantification of the indicated conditions with ≥5 γ-H2AX TIFs in (D). Data show the mean from 150 nuclei analyzed per cell line.

Both telomere hyper-elongation and TIFs were not observed in early-generation but only in late-generation *Pot1b^−/−^* cells (Figure 1B–D, Figure 4A, B, Supplementary Figure S5a), suggesting that these phenotypes are likely associated to each other. Telomerase recruitment in yeast is promoted through activation of homologues of the ATR and ataxia-telangiectasia mutated (ATM) DDR pathways (69,70,72). ATR and ATM pathways are critical for facilitating telomerase recruitment in mammalian systems (39,68). We postulated that the elevated DDR observed in G7 *Pot1b^−/−^* cells (Figure 3C, D) led to increased telomerase recruitment. To test this notion, we repressed the DDR at telomeres of G7 *Pot1b^−/−^* cells by overexpressing POT1b, and POT1ba. Overexpression of POT1b repressed TIF formation in G7 *Pot1b^−/−^* cells but did not lead to a decrease in telomerase recruitment (Figure 6A–C, Supplementary Figure S7a, b). However, the C-terminus of full-length POT1b would not only repress the DDR at telomeres but would also promote telomerase recruitment through its interaction with TPP1 (23), likely confounding any effect repression of DDR by full-length POT1b might have on telomerase recruitment. Overexpression of POT1ba in G7 *Pot1b^−/−^* cells repressed TIF formation and decreased telomerase recruitment by approximately 28% (Figure 6A–C, Supplementary Figure S7a, b). To interrogate the role of ATR-dependent DDR in telomerase recruitment more directly, we inhibited ATR in G7 *Pot1b^−/−^* cells using ATRi AZ20 and measured telomerase recruitment. ATRi decreased TIF formation and telomerase recruitment rates by ∼26%, further suggesting that the DDR activation is required to promote telomerase recruitment to telomeres in G7 *Pot1b^−/−^* cells (Figure 6A–C).

ATR/ATM pathways are critical for telomerase complex assembly (39). Potentially, inhibition of telomerase recruitment by ATRi could be the result of decreased telomerase complex biogenesis instead of telomerase recruitment to telomeres. Similarly, POT1ba might be inhibiting telomerase recruitment through similar mechanisms as POT1a (Figure 3C, D) and not through repression of TIFs (23). We therefore combined POT1ba + ATRi treatments to determine if these conditions are epistatic, resulting in diminished telomerase recruitment. When compared to POT1ba + DMSO (28% decrease) or Vector + ATRi (26% decrease), POT1ba + ATRi did not further reduce telomerase recruitment, with a 29% decrease in TR/telomere colocalizations per nucleus compared to Vector + DMSO (Figure 6A–C). This result suggests that both POT1ba and ATRi treatment inhibited telomerase recruitment through the same pathway. Similarly, POT1b^WT^ overexpression prevents ATRi inhibitory effect on telomerase recruitment as POT1b^WT^ + ATRi did not decrease telomerase recruitment compared to POT1b^WT^ + DMSO (Figure 6A–C). Given that POT1b^WT^ represses TIFs, this finding is consistent with the hypothesis that elevated ATR-dependent DDR at telomeres is promoting telomerase recruitment in G7 *Pot1b^−/−^* cells. To confirm that it is the repression of the ATR-dependent DDR at telomeres that is reducing telomerase recruitment, we measured the effect of POT1ba and ATRi in G3 *Pot1b^−/−^* cells without robust DDR activation at telomeres (Figure 6D–F, Supplementary Figure S7c, d). Neither POT1ba nor ATRi decreased telomerase recruitment in G3 *Pot1b^−/−^* cells, revealing that increased telomerase recruitment to dysfunctional telomeres in G7 *Pot1b^−/−^* cells is responsible for its telomere hyper-elongation phenotype (Figure 6D–F, Supplementary Figure S7c, d).

### Telomerase is required for DNA damage at telomeres in late-generation *Pot1b^−/−^* cells

**Figure 7.**
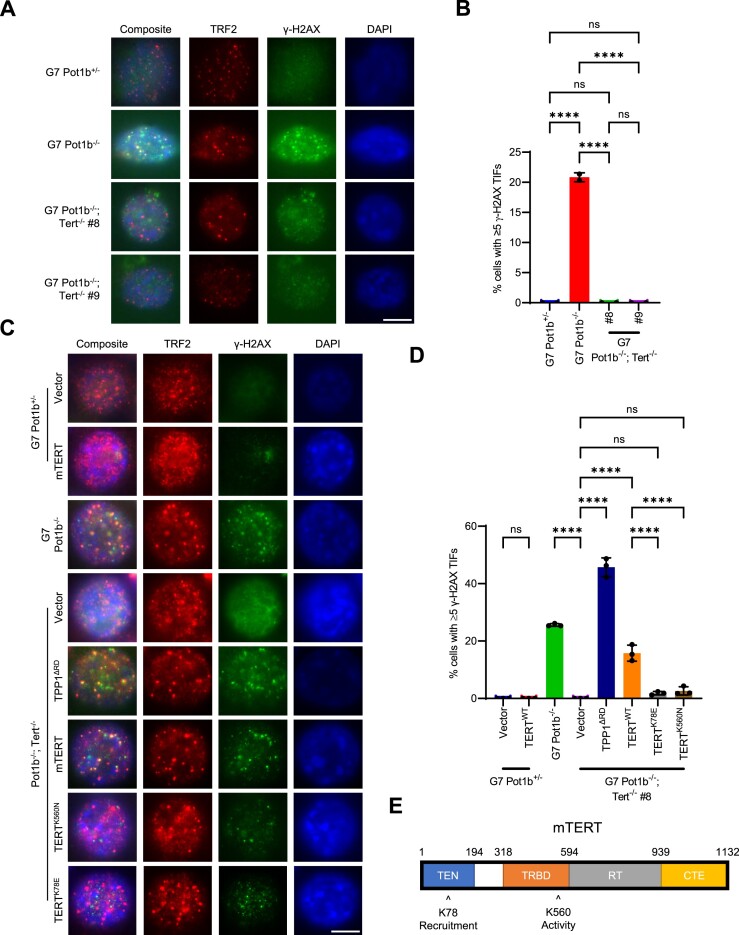
Telomerase activity is required to promote telomeric damage in late-generation *Pot1b^−/−^* cells. (**A**) Representative images of γ-H2AX TIFs in the indicated cell lines. Cells were immunostained with γ-H2AX antibody (green), TRF2 antibody (red) and DAPI to stain nuclei (blue). Scale bar: 5μm. (**B**) Quantification of the indicated cell lines with ≥5 γ-H2AX TIFs in (A). Data show the mean ± standard deviation from two independent experiments in which 150 nuclei were analyzed per cell line. *P*-values are shown and generated from one-way ANOVA analysis followed by Tukey's multiple comparison. (**C**) Representative images of γ-H2AX TIFs in the indicated cell lines expressing the indicated constructs. Cells were immunostained with γ-H2AX antibody (green), TRF2 antibody (red) and DAPI to stain nuclei (blue). Scale bar: 5μm. (**D**) Quantification of the indicated cell lines with ≥ 5 γ-H2AX TIFs in (C). Data show the mean ± standard deviation from three independent experiments in which 150 nuclei were analyzed per cell line. *P*-values are shown and generated from one-way ANOVA analysis followed by Tukey's multiple comparison. (**E**) Schematic of mTERT showing the K78 and K560 amino acids.

An important question to address is how damaged telomeric DNA is generated in G7 *Pot1b^−/−^* cells. POT1b loss would prevent CST recruitment to telomeres, leading to elevated telomere replication-dependent defects detected as fragile telomeres. However, telomere fragility was indistinguishable between *Pot1b^+/−^* and G7 *Pot1b^−/−^* cells (Supplementary Figure S4a, b). Furthermore, POT1b^WT^ overexpression, which readily repressed TIF formation, did not decrease the number of fragile telomeres (Figure 5A, B, Supplementary Figure S4a, b). These results indicate that replication stress is unlikely to be the source of telomere damage. Another possible source of telomere damage is telomeric repeat-containing RNA (TERRA), which promotes RPA displacement and POT1 binding following telomere replication (75). However, TERRA was not detectable by RNA-FISH in late-generation *Pot1b^+/−^* and *Pot1b^−/−^* cells (Supplementary Figure S4d, e). Another potential source of damaged telomeric DNA could arise from aberrant telomeric repeats generated by telomerase (76,77) as it hyper-elongates telomeres. To address this hypothesis, we deleted *Tert* using CRISPR/Cas9. While the parental G7 *Pot1b^−/−^* cells displayed ≥5 γ-H2AX TIFs in ∼25% of nuclei, deletion of *Tert* completely abolished the formation of γ-H2AX-positive TIFs in two independent CRISPR/Cas9 *Tert* KO clones (Figure 7A, B). TERT^WT^ overexpression in G7 *Pot1b^−/−^; Tert^−/−^* cells readily restored γ-H2AX TIFs (Figure 7C, D, Supplementary Figure S8a–d), revealing that TIF formation in late-generation *Pot1b^−/−^* cells requires telomerase. Overexpression of TPP1^ΔRD^ in G7 *Pot1b^−/−^; Tert^−/−^* cells lead to robust TIF formation, suggesting that DDR pathways are not impaired by *Tert* deletion (Figure 7C, D). To understand the mechanism through which telomerase promotes telomeric damage in G7 *Pot1b^−/−^* cells, we overexpressed TERT^K78E^ (which disrupts telomerase recruitment to telomeres but not catalytic activity) (78,79) and TERT^K560N^ (which abolishes telomerase catalytic activity but not recruitment) (79,80) in G7 *Pot1b^−/−^; Tert^−/−^* cells (Figure 7C–E, Supplementary Figure S8a–d). Neither TERT^K78E^ nor TERT^K560N^ overexpression restored TIF formation in G7 *Pot1b^−/−^; Tert^−/−^* cells (Figure 7C–E, Supplementary Figure S8a–d), indicating that telomerase recruitment to telomeres and its catalytic activity are both required for TIF formation. Our data suggests that the telomeric repeats synthesized by telomerase in G7 *Pot1b^−/−^* cells are recognized as damaged DNA.

### The telomeric 3′ overhangs of G7 *Pot1b^−/−^* sarcomas contain G-quadruplexes

**Figure 8.**
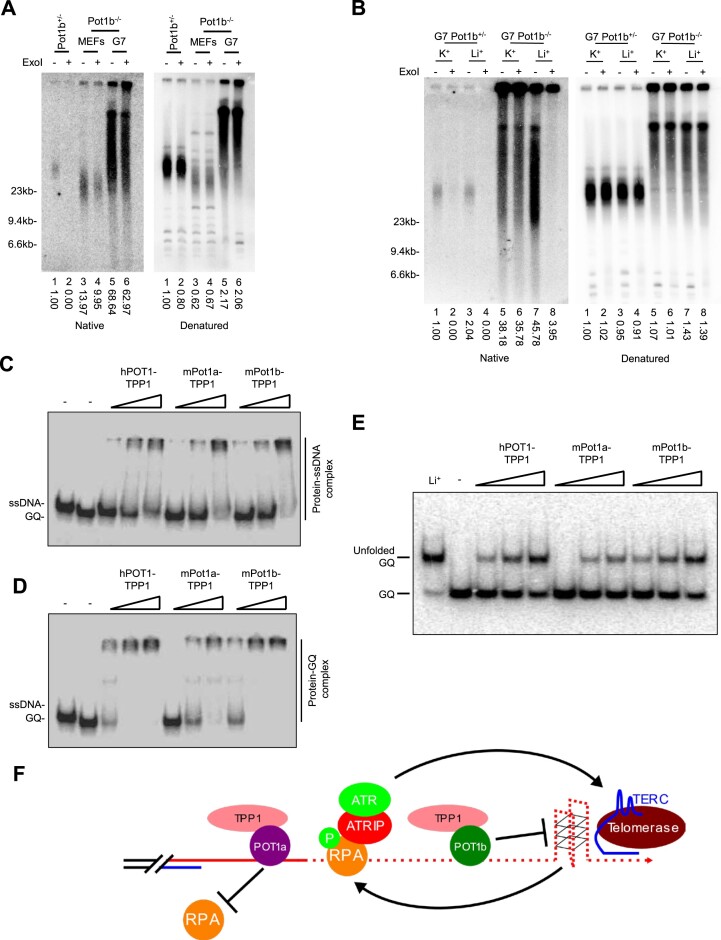
Telomeric overhangs in late-generation *Pot1b^−/−^* cells contain G-quadruplexes normally resolved by POT1b. (**A**) TRF Southern blot of DNAs derived from the indicated cell lines detected by in-gel hybridization with γ-^32^P-(CCCTAA)_4_ telomere probe. DNA was treated with and without *E. coli* Exonuclease I (ExoI). Relative signals for G-overhangs (native gel) and total telomere lengths (denatured gel) are indicated, with telomere signals set to 1.0 for *Pot1b^+/−^* MEFs without ExoI. Molecular weight markers as indicated. (**B**) TRF Southern blot detection of G-overhang (native gel) and total telomere length (denatured gel) of G7 *Pot1b^+/−^* and G7 *Pot1b^−/−^* DNA hybridized with γ-^32^P-(CCCTAA)_4_ telomere probes. DNA was treated with and without *E. coli* Exonuclease I in the presence of 150 mM KCl or 150 mM LiCl as indicated. Numbers indicate relative G-overhang and total telomere signals, with telomere signals set to 1.0 for G7 *Pot1b^+/−^* without ExoI in 150 mM KCl. Molecular weight markers as indicated. (**C**) Native gel analysis of 50nM single-stranded 5′-(TTAGGG)_3_–3′ oligonucleotide with 0, 50, 100 and 200 nM of hPOT1, POT1a or POT1b. (**D**) Native gel analysis of 50nM G-quadruplex forming 5′-GGG(TTAGGG)_3_–3′ oligonucleotide with 0, 50, 100 and 200 nM of hPOT1, POT1a or POT1b. (**E**) Native gel analysis of 50nM G-quadruplex forming 5′-GGG(TTAGGG)_3_–3′ oligonucleotide with 0, 50, 100 and 200 nM of hPOT1, POT1a or POT1b followed by proteinase K treatment. (**F**) Schematic of proposed model of GQ formation and telomere hyper-elongation in the absence of POT1b.

We used native and denaturing TRF Southerns to characterize the nature of the telomeric DNA damage in G7 *Pot1b^−/−^* cells. G7 *Pot1b^−/−^* telomeres possess only single-stranded TTAGGG overhangs while ss CCCTAA-overhangs were not detected (Supplementary Figure S9a). Surprisingly, the ss G-overhang in G7 *Pot1b^−/−^* cells was completely resistant to the *E. coli* 3′-5′ Exonuclase I (ExoI) digestion (Figure 8A, Supplementary Figure S9b), suggesting that it contains secondary structures that prevents ExoI hydrolysis. The G-rich telomeric strand is known to fold into G-quadruplexes (GQ), four-stranded DNA helices formed from stacks of square-planar arrays known as G-quartets which are stabilized by guanine hydrogen bonding and monovalent cations such as K^+^ or Na^+^ but destabilized by Li^+^ cations (81,82). GQs inhibit *E. coli* ExoI mediated digestion of telomeric DNA *in vitro* (83). We postulated that the long ss G-overhangs in hyper-elongated G7 *Pot1b^−/−^* telomeres might be prone to form GQ structures inhibitory to ExoI digestion. To test this hypothesis, we performed ExoI digestion in a GQ-stabilizing (150 mM KCl) or a GQ-destabilizing (150 mM LiCl) buffer (Figure 8B, Supplementary Figure S9c). We again observed a complete resistance to ExoI digestion in DNA from the G7 *Pot1b^−/−^* cells treated with 150 mM KCl (Figure 8B, Supplementary Figure S9c). However, treatment of G7 *Pot1b^−/−^* telomeres with 150 mM LiCl allowed ExoI to almost completely digest the ss G-rich overhang (Figure 8B, Supplementary Figure S9c). We confirmed this phenotype in an independent *Pot1b^−/−^; p53^−/−^* sarcoma where telomeric overhangs were also resistant to ExoI digestion in the presence of KCl but sensitized with LiCl (Supplementary Figure S9d, e). Previous *in vitro* and structural works have suggested that GQs form during telomerase activity (84,85). To evaluate if the increased telomerase elongation is contributing to GQ formation, we performed ExoI digestion in two G7 *Pot1b^−/−^; Tert^−/−^* cell lines we had generated using CRISPR/Cas9. While the telomeric overhangs in the parental line were nearly completely resistant to ExoI digestion, *Tert* KO sensitized the ss G-rich overhang by ∼37% in both CRISPR/Cas9 clones (Supplementary Figure S9f, g). Overexpressing TERT in G7 *Pot1b^−/−^; Tert^−/−^* cells reduced the sensitivity to ExoI hydrolysis (Supplementary Figure S9f, g). These data suggest that the increased telomerase-mediated telomere elongation is promoting GQ formation in the telomeric overhang. However, the ss G-rich overhang is still partially resistant to ExoI digestion in G7 *Pot1b^−/−^; Tert^−/−^* DNA suggesting that GQ resolution is still impaired even in the absence of telomerase.

### POT1b, but not POT1a, resolves telomeric G-quadruplexes

The Cech laboratory has shown previously that hPOT1 can resolve GQs *in vitro* (46). To determine whether POT1a or POT1b can promote GQ unfolding in G7 *Pot1b^−/−^* cells, we evaluated ExoI sensitivity of overhangs expressing POT1a^WT^, POT1b^WT^, POT1ab, POT1ba and hPOT1. Expression of POT1b^WT^ but not POT1a^WT^ increased G-overhang sensitivity to ExoI digestion, and this property was dependent upon the presence of a WT POT1b N-terminus (Supplementary Figure S9h, i). Additionally, hPOT1 overexpression in G7 *Pot1b^−/−^* cells also increased G-overhang sensitivity to ExoI, revealing functional conservation between hPOT1 and POT1b (Supplementary Figure S9h, i). To further confirm that POT1b is able to unfold GQs, we purified hPOT1 with hTPP1, POT1a^WT^ and POT1b^WT^ with mTPP1 proteins and performed an electrophoretic mobility shift assay (EMSA), using telomeric oligonucleotides (TTAGGG)_3_ or GGG(TTAGGG)_3_ (46). GGG(TTAGGG)_3_ but not (TTAGGG)_3_ is able to fold into a GQ structure, allowing it to migrate faster by native gel electrophoresis despite being a longer oligonucleotide (Figure 8C, D) (46). While all three POT1 proteins readily formed complexes with (TTAGGG)_3_ (Figure 8C), POT1a displayed a diminished ability to interact with GGG(TTAGGG)_3_ GQs. In contrast, both hPOT1 and POT1b readily formed complexes with GGG(TTAGGG)_3_ (Figure 8D). Finally, we asked if POT1b can unfold GQs. We incubated POT1 proteins at various concentrations with GGG(TTAGGG)_3_ GQs, removed proteins with SDS and Proteinase K treatment and performed native gel electrophoresis to detect GQs and unfolded GQs. While 50 nM of hPOT1 and POT1b were able to unfold GQs, POT1a was unable to unfold GQs at this protein concentration. Compared to hPOT1 and POT1b, POT1a was also less efficient at unfolding GQs at all protein concentrations (Figure 8E). These results revealed that POT1b, but not POT1a, can efficiently interact with and unfold GQs similar to hPOT1.

## DISCUSSION

Serial transplantation of *Pot1b*^−/−^ sarcomas into SCID mice has uncovered a role for POT1b in the regulation of telomere elongation and repression of an ATR-dependent DDR. Our speculative model is shown in Figure 8F. Telomere shortening in mid-generation *Pot1b^−/−^* cells leads to activation of a DDR, promoting telomerase recruitment and telomere hyper-elongation. Increased telomere hyper-elongation leads to GQ accumulation in the telomeric overhang that would otherwise be resolved by POT1b. Without POT1b, the GQ structures are unfolded by RPA, leading to activation of an aberrant ATR-dependent DDR. The ATR pathway then promotes further telomerase recruitment, resulting in a positive feed-back loop to generate further telomere hyper-elongation and telomere DDR activation. We demonstrate that the repression of this DDR at telomeres is conserved between POT1b and hPOT1, suggesting that similar mechanisms may underly the phenotypes observed in tumors harboring hPOT1 cancer mutations.

### Telomere elongation in POT1 mutant cancers

Next generation sequencing identified hPOT1 mutations in a wide variety of cancers, making hPOT1 the most frequently mutated shelterin component in human cancers (27,28,30,36,86). However, given the diverse functions that hPOT1 plays in telomere regulation, our understanding of its functions in healthy and diseased tissues remain incomplete. Two phenotypes commonly associated with cancer hPOT1 mutations are telomere elongation and genomic instability (25). Through long-term serial passaging of *Pot1b^−/−^* sarcomas in SCID mice, we were able to generate late-generation *Pot1b^−/−^* cell lines displaying both telomere hyper-elongation as well as an ATR-dependent DDR at telomeres, thereby mimicking the most prominent phenotypes observed in cancers with hPOT1 mutations. We provide experimental evidence in *Pot1b^−/−^* cells that the telomere hyper-elongation and DDR activation are inextricably linked, with each phenotype promoting the other. POT1b has a role distinct from POT1a in preventing ATR activation at telomeres and is specifically required to resolve GQs that are produced during telomere elongation by telomerase (Figure 8F).

### Increased telomerase recruitment and telomere hyper-elongation mediated by ATR activation

POT1b is critical for telomere length maintenance by recruiting the CST complex to modulate C-strand fill-in and promoting telomerase recruitment to the G-strand (16,23). Consequently, POT1b loss has been associated with accelerated telomere shortening rather than telomere elongation (21,22). Consistent with this notion, early generation *Pot1b^−/−^* sarcomas also undergo progressive telomere shortening (Supplementary Figure S1b, c). However, after undergoing extensive passaging *in vivo*, telomere shortening in *Pot1b^−/−^* sarcomas is reversed and telomerase-mediated telomere hyper-elongation phenotype begins to dominate in two independently generated *Pot1b^−/−^* sarcoma serial transplantations (Figure 1B–D, Supplementary Figure S1d, e). Because telomere hyper-elongation is only observed after extensively passaging of *Pot1b^−/−^* cells *in vivo*, we speculate that global telomere shortening is critical to begin the process of telomere hyper-elongation. Previous work has suggested that the shortest telomeres are preferentially elongated by telomerase (87–90). Consequently, it is likely that a few shortened telomeres of mid-generation *Pot1b*^*−/−*^ cells promote telomerase recruitment. Elongation of the shortest telomeres is well characterized in *S. cerevisiae* which requires the yeast ATM homolog Tel1p, suggesting a role for the DDR to promote telomerase recruitment to critically shortened telomeres (66,67,71). Extensive work in both fission and budding yeast also demonstrates that Mec1p/Rad3p, yeast homologs of ATR, share functional redundancy with Tel1p and are both critical in facilitating telomerase recruitment to telomeres (69,70,72,91). ATM and ATR are required for telomerase recruitment in mammalian cells, suggesting functional conservation of DDR regulation of telomerase-mediated telomere length maintenance (39,68). Therefore, critically short telomeres may activate DDR pathways as an initiating event, leading to increased telomerase recruitment in mid-generation *Pot1b^−/−^* sarcomas. However, we cannot exclude the possibility that these *Pot1b^−/−^* sarcomas may have accumulated somatic mutations during passaging that contribute to telomere elongation. Once critically short telomeres elongate in a POT1b-proficient setting, shelterin function would normally be restored to repress the DDR and maintain telomere lengths. However, we demonstrate that telomerase activity in the absence of POT1b produces a DDR at telomeres (Figure 7). This DDR then promotes telomerase recruitment leading to further telomere elongation in a positive feedback loop, resulting in sustained telomere hyper-elongation (Figure 8F). We speculate that early generation *Pot1b*^*−/−*^ cells have not entered this positive feedback loop as the DDR is substantially weaker, leading to continued telomere shortening.

Previous investigations of hPOT1 and POT1a functions revealed that disruption of their OB-folds leads to increased telomere replication stress and telomere elongation (33,35,39,92). Increased replication stress, ATR activation and rapid telomere elongation are also reminiscent of the phenotypes observed with TRF1 loss (93). However, we are unable to detect any signs of replication stress in late-generation *Pot1b^−/−^* cells (Figure 1B–D, Supplementary Figure S4a, b). Our data suggest that an ATR-dependent DDR alone could promote telomerase-mediated telomere hyper-elongation without requiring activation of replication stress at telomeres.

### POT1b represses ATR activation and GQ formation at telomeres

Previous reports suggest that POT1b does not play a significant role in protecting ss-telomeric DNA from activating a DDR (9,22). However, our data reveal a uncharacterized role for POT1b in protecting telomeres from activating a DDR at telomeres (Figure 5A, B). Early-generation *Pot1b^−/−^* cells do not have a detectable DDR at telomeres and TIF formation only appeared after extensive *in vivo* passaging (Figure 4A, B). Activation of this DDR in the absence of POT1b has never been characterized. We also discovered that telomerase activity at telomeres is required for TIF formation in the absence of POT1b (Figure 7A–E). Our data suggest that telomerase activity is generating the GQs (Supplementary Figure S9f, g) that are recognized as DNA damage by RPA, which explain why telomerase is required for DDR activation. *In vitro* experiments have demonstrated that GQs form within actively extending telomeres (84). Recent structural data supports this finding by revealing that hPOT1 and telomerase form a cavity that is sterically large enough to accommodate folding of telomeric DNA into a GQ (85). This suggests that nascent repeats polymerized by telomerase form GQs which then must be unfolded by hPOT1 (84). POT1b but not POT1a is preferentially incorporated into the telomerase/TPP1/POT1b/telomere complex to initiate telomere elongation, as POT1b promotes telomerase recruitment to telomeres (23). POT1b would then be in a prime position to resolve GQ structures that fold as telomerase synthesizes new telomeric repeats. GQs have been strongly implicated to be obstacles for replication fork progression and are a source for increased replication stress (94,95). However, late-generation *Pot1b^−/−^* cells do not display increased fragile telomere and show no overt signs of replication stress (Supplementary Figure S4a, b). Since GQs generated by telomerase would be contained within the 3′ telomeric overhang, it is possible that the replication machinery never reaches the GQs as the eukaryotic replicative 3′-5′ helicase, minichromosome maintenance (Mcm2-7) complex, may fall off the 5′ end at the ds-ss telomere junction (96).

In the absence of POT1b, unresolved GQs in telomeres may be unfolded by other proteins such as RPA, a known GQ resolving protein complex (97). In this setting, GQs would aberrantly activate an ATR-mediated DDR. Consequently, POT1b's resolution of GQs may be the mechanism through which POT1b is repressing the activation of a DDR in telomeres of late-generation *Pot1b^−/−^* cells (Figure 5). This model is also consistent with POT1a's inability to repress TIF formation in G7 *Pot1b^−/−^* sarcomas (Figure 5), as POT1a is less proficient at resolving GQ than POT1b (Figure 8D, E). Additionally, POT1b's repression of TIF formation in G7 *Pot1b^−/−^* cells requires its N-terminus (Figure 5C–E), which contains the ss-telomeric DNA binding OB folds critical for GQ resolution (Supplementary Figure S9h, i). Interestingly, the CST complex also has GQ resolving properties (98) and it is possible that POT1b recruitment of the CST complex (16) may further facilitate GQ unfolding. However, our data reveal that POT1b alone is fully capable to unfold GQs (Figure 8D, E) and repress TIF formation (Figure 5A, B).

Cancer-associated mutations in hPOT1 have been linked to telomere elongation and genomic instability (25), similar to the phenotypes observed in late-generation *Pot1b^−/−^* sarcomas. However, the underlying mechanisms that drive telomere elongation and genomic instability in hPOT1-mutant cancers remain poorly understood. The characterization of late-generation *Pot1b^−/−^* sarcomas can serve as a valuable model to shed light on the impact of hPOT1 cancer-associated mutations. Our findings reveal that both hPOT1 and POT1b share a conserved ability to unfold GQs and repress TIFs (Figures 5D–E, 8C–E). Therefore, it is plausible that some hPOT1 cancer mutations disrupt the ability to resolve GQ structures at telomeres, leading to activation of the ATR pathway and promoting telomerase recruitment to the telomeres. However, the influence of hPOT1 cancer mutations on GQ resolution has yet to be characterized. It is important to note that other mechanisms may also be involved, as evidenced by reports of telomere elongation in heterozygous hPOT1^WT/Q623H^ stem cells without detectable TIFs (34). Furthermore, it remains to be determined whether mutant hPOT1 exhibit a similar pattern of initial telomere shortening followed by elongation, as observed in the *Pot1b^−/−^* sarcomas (Figure 1B–D). Future investigations are needed to unravel the molecular consequences of hPOT1 cancer mutations and their impact on telomere length maintenance mechanisms necessary to promote cancer progression.

## Supplementary Material

gkad648_Supplemental_File

## Data Availability

All data are incorporated into the article and its online supplementary material.

## Notes

Present address: Taylor Takasugi, Department of Laboratory Medicine, Yale University School of Medicine, New Haven, CT 06520, USA.

Present address: Sandy Chang, Department of Laboratory Medicine, Yale University School of Medicine, New Haven, CT 06520, USA.
