# Supplementary material for: Pot1b −/− tumors activate G-quadruplex-induced DNA damage to promote telomere hyper-elongation
Source: Nucleic Acids Res. 2023 Aug 10;51(17):9227–47. doi: 10.1093/nar/gkad648 (PMC10516629; doi:10.1093/nar/gkad648)
Supplement: gkad648_Supplemental_File [file gkad648_supplemental_file.pdf]

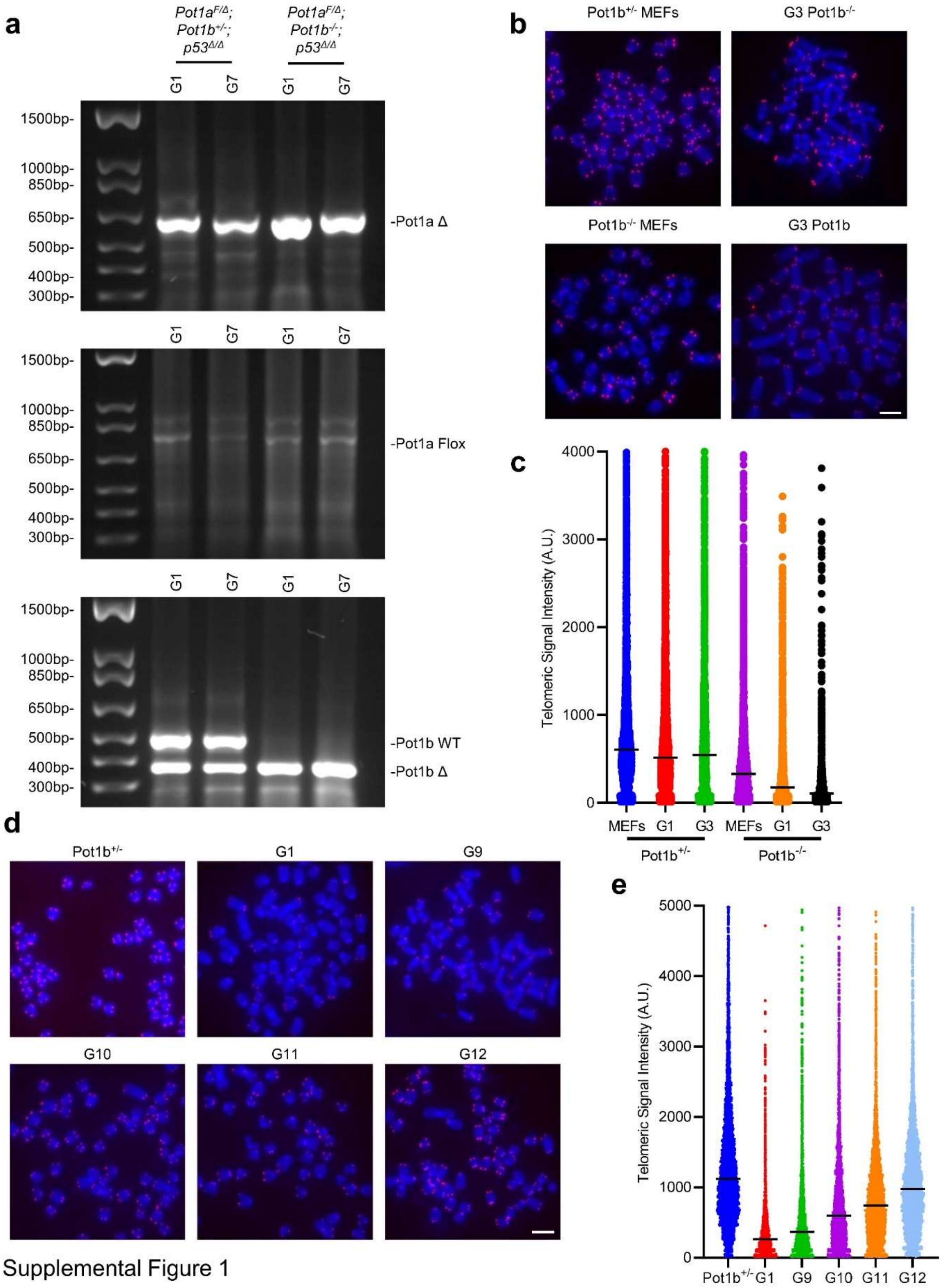

Supplemental Figure 1

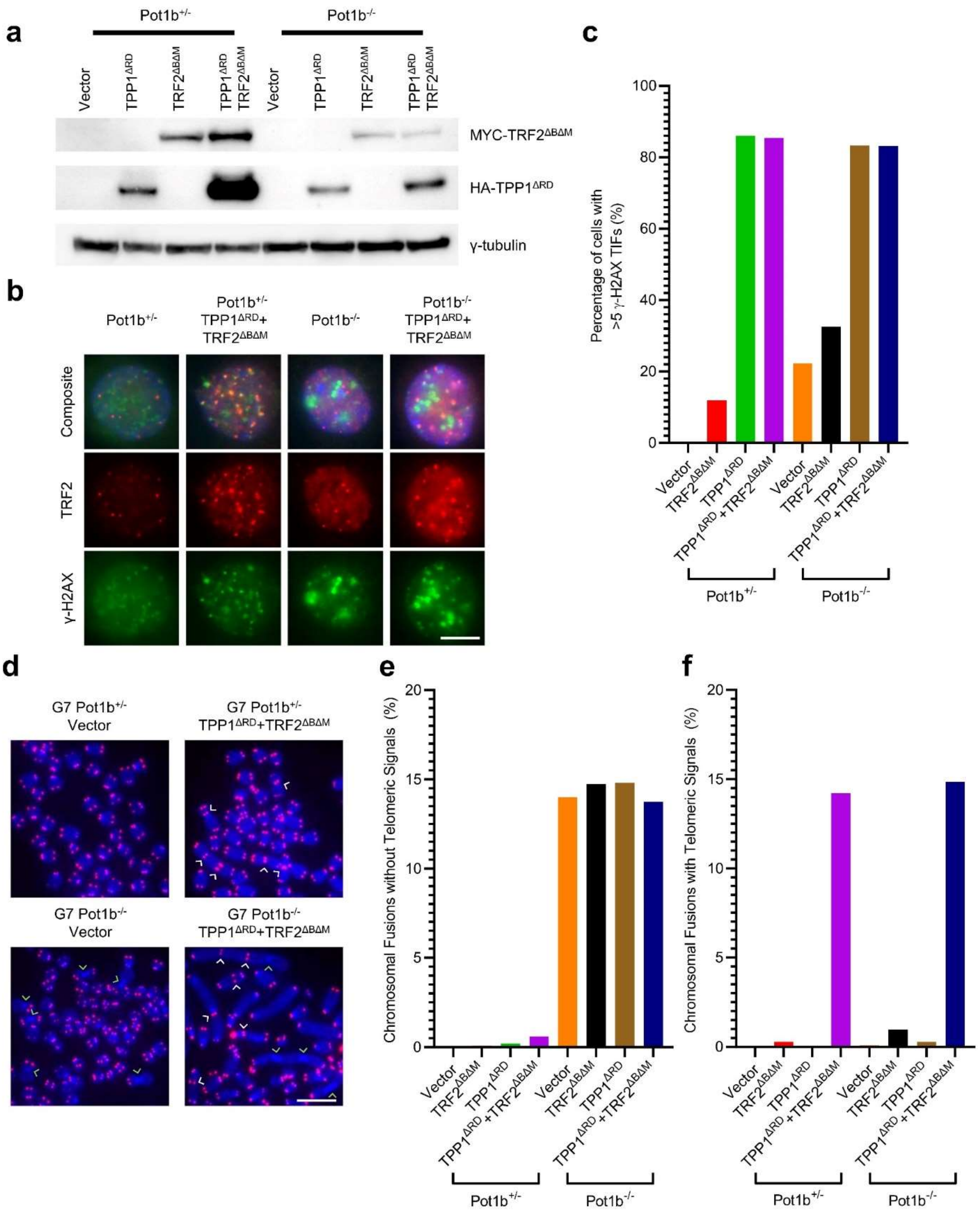

Supplemental Figure 2

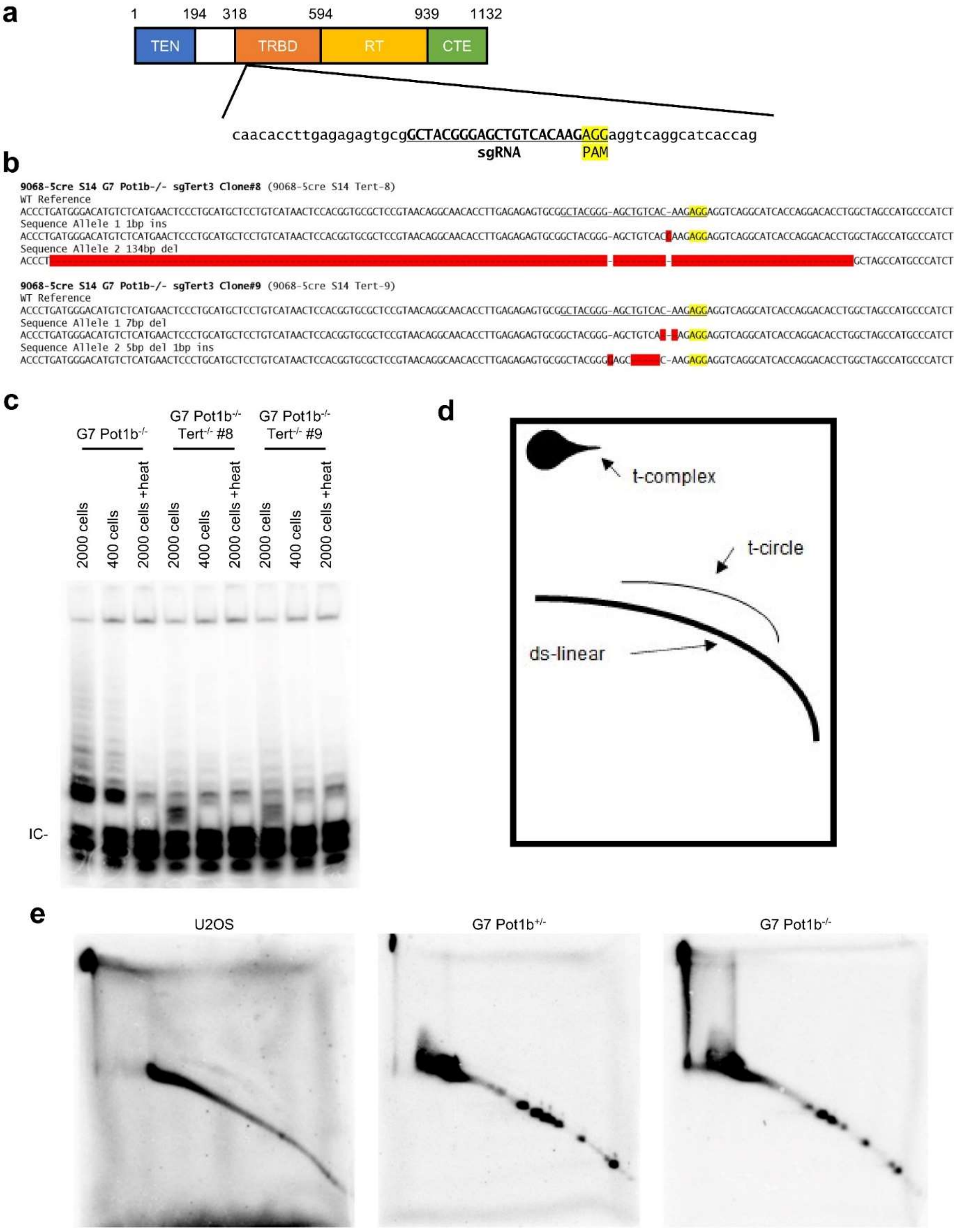

Supplemental Figure 3

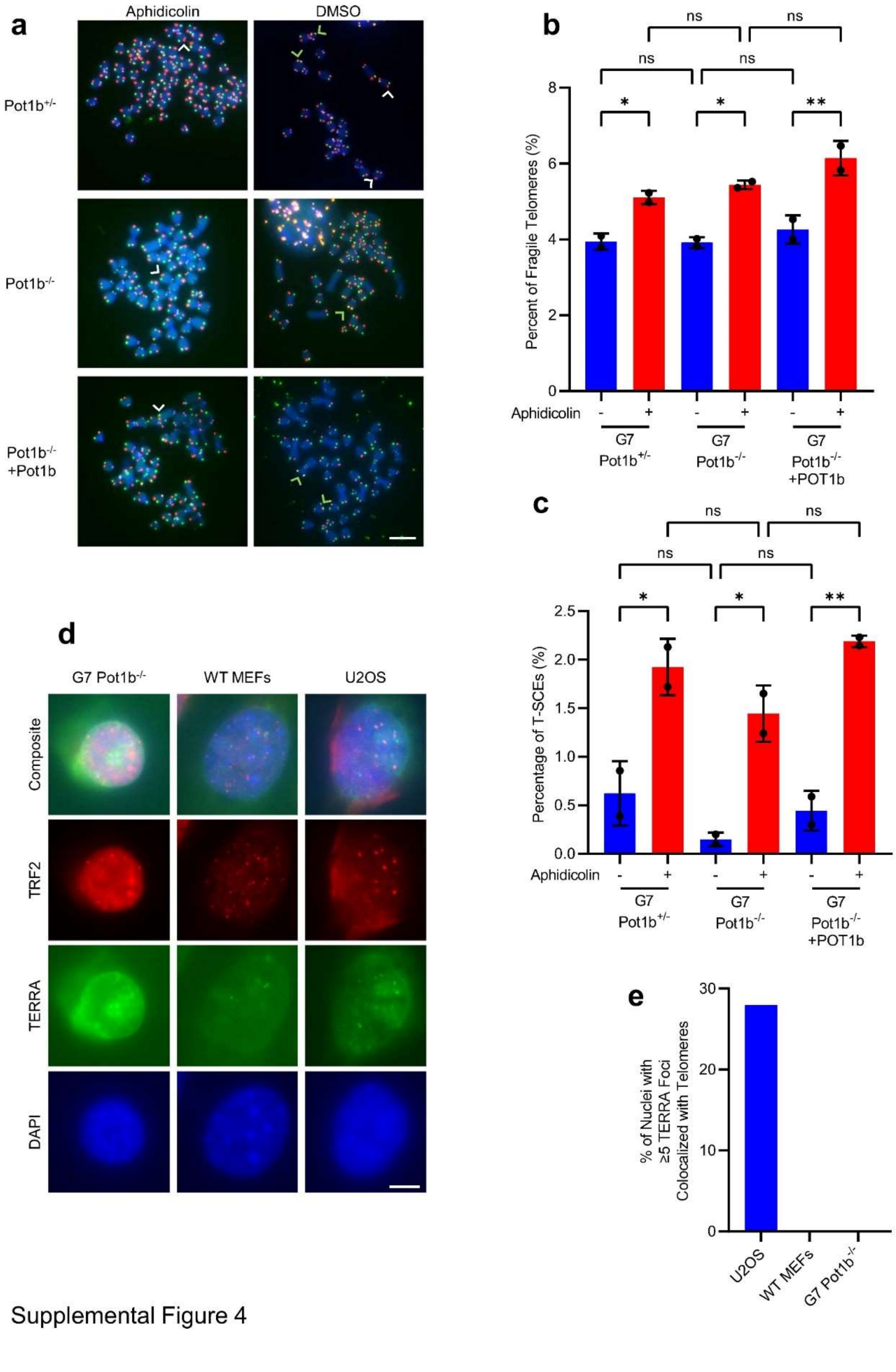

Supplemental Figure 4

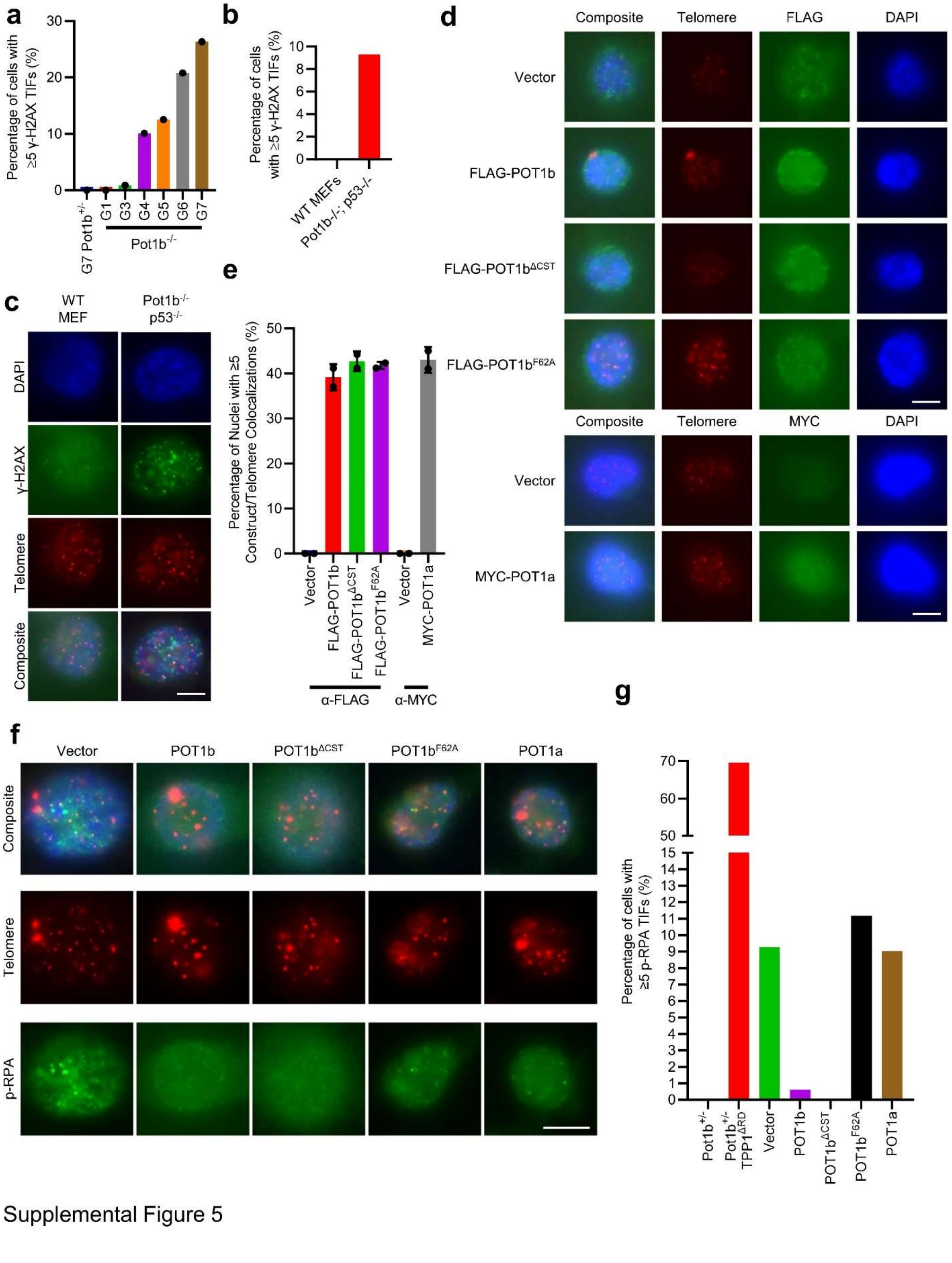

Supplemental Figure 5

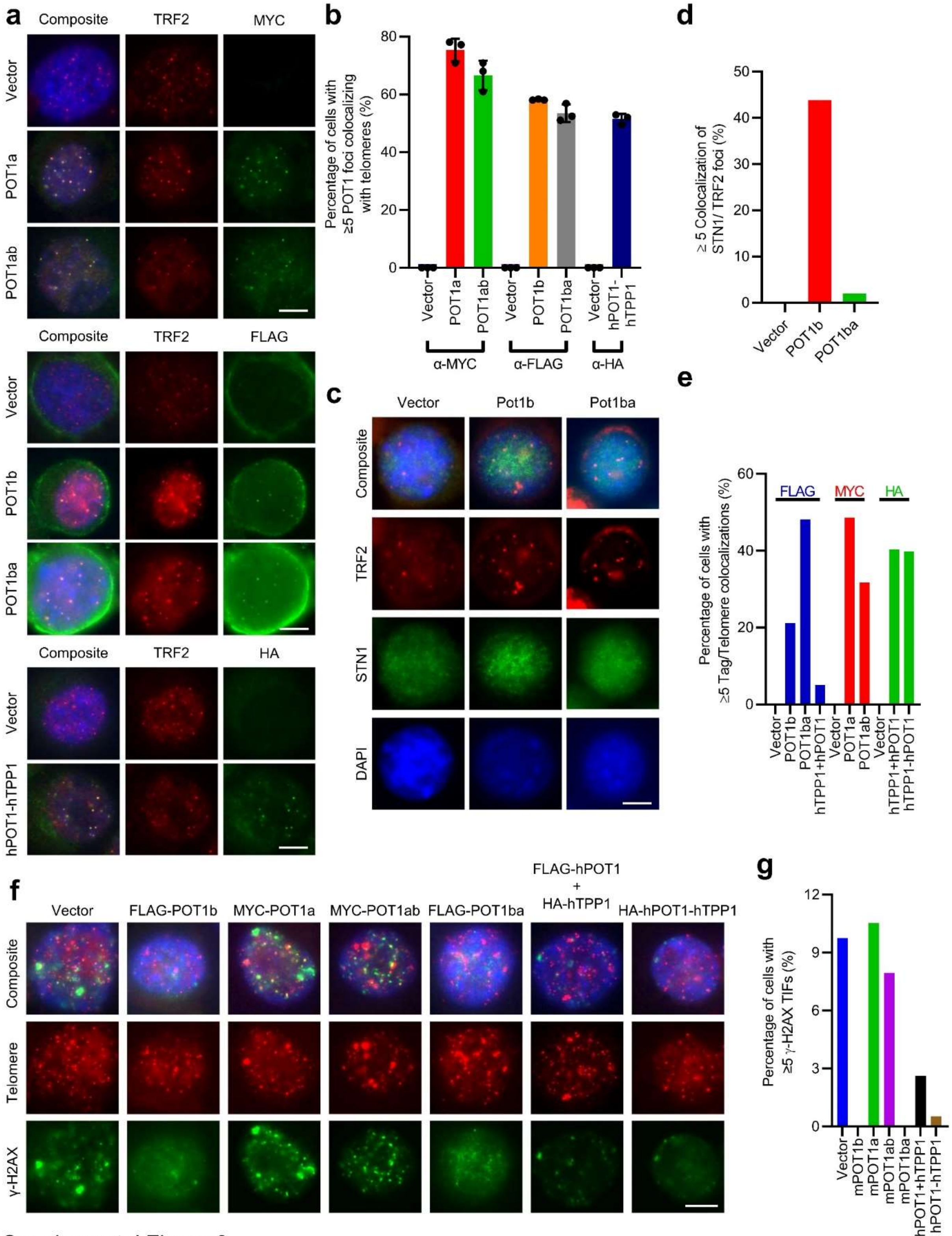

Supplemental Figure 6

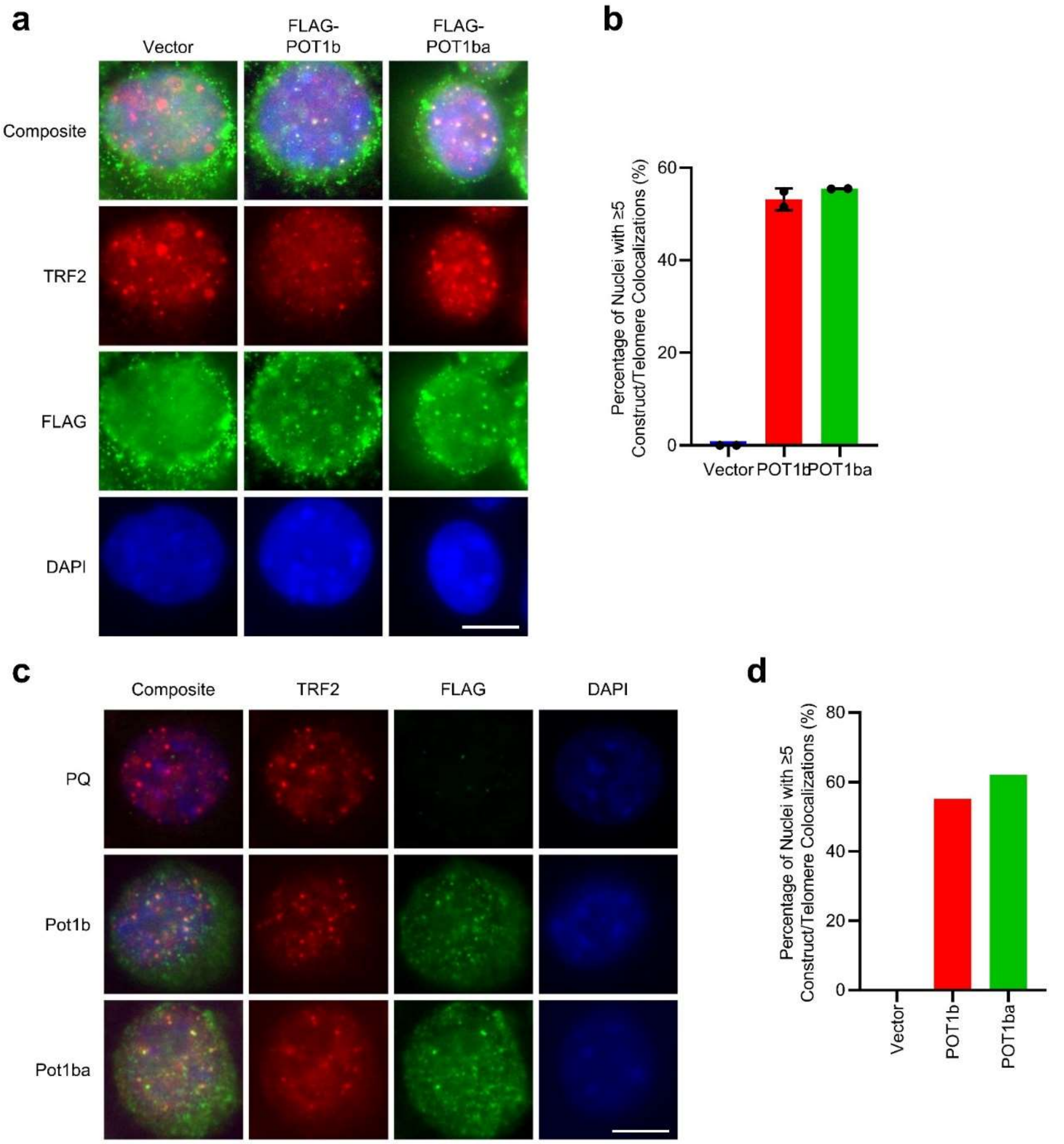

Supplemental Figure 7

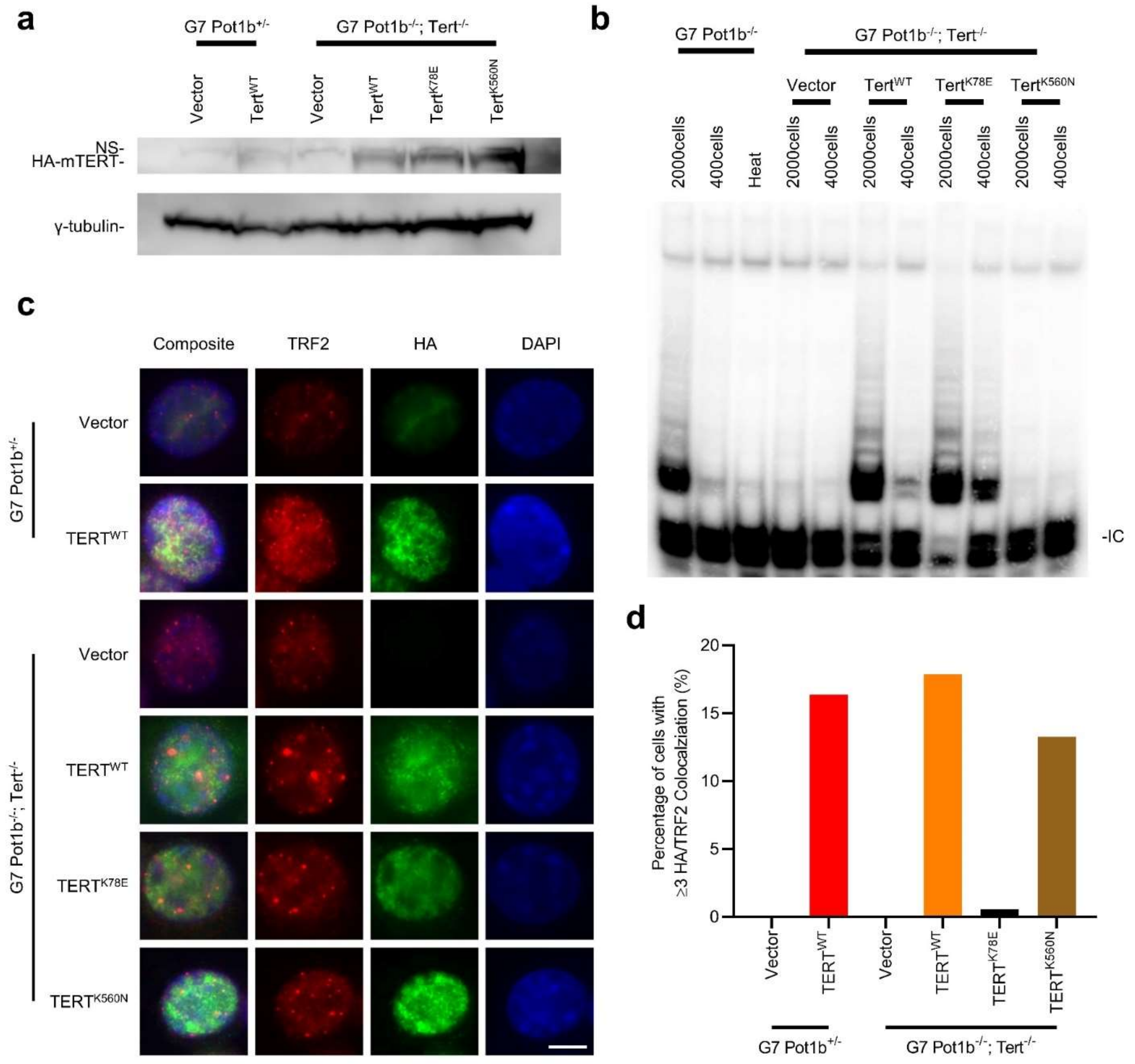

Supplemental Figure 8

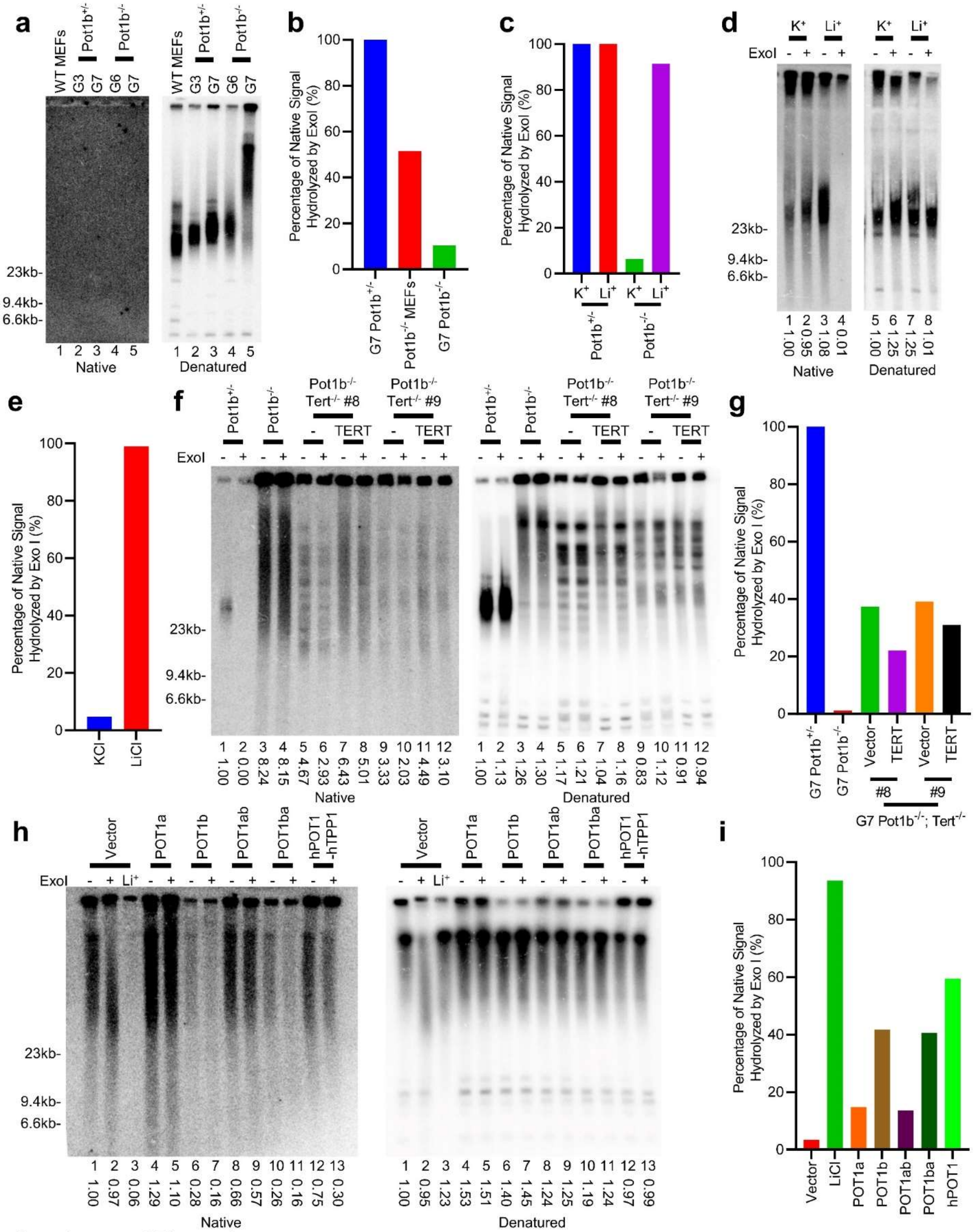

Supplemental Figure 9

Supplemental Figure Legends

Supplemental Figure 1. Telomere lengths in *Pot1b$^{-/-}$* sarcomas. a. PCR genotyping of G1 and G7 *Pot1b$^{+/-}$* and *Pot1b$^{-/-}$* cell lines. **b.** Representative images of metaphase spreads of the indicated cell lines visualized with PNA-FISH probe Cy3-OO-$(CCCTAA)_3$ and DAPI. Scale bar: 5µm. **c.** Q-FISH analysis showing the median telomeric signal intensity from metaphases in (a). 30 metaphases were scored per cell line. **d.** Representative images of metaphase spreads of the indicated generations of independently generated *Pot1b$^{-/-}$; p53$^{-/-}$* serial transplantation sarcomas visualized with PNA-FISH probe Cy3-OO-$(CCCTAA)_3$ and DAPI. Scale bar: 5µm. **e.** Q-FISH analysis showing the median telomeric signal intensity from metaphases in (d). 30 metaphases were scored per cell line.

Supplemental Figure 2. Disrupting shelterin function in late-generation *Pot1b$^{+/-}$* and *Pot1b$^{-/-}$* cells. a. Western blot of G7 *Pot1b$^{+/-}$* and *Pot1b$^{-/-}$* cells expressing the indicated construct. **b.** Representative images of γ-H2AX TIFs in the indicated cell lines. Cells were immunostained with γ-H2AX antibody (green), mTRF2 antibody (red) and DAPI to stain nuclei (blue). Scale bar: 5µm. **c.** Quantification of 150 nuclei from the indicated cell lines with ≥5 γ-H2AX TIFs in (b). **d.** Representative images of metaphase spreads of the indicated cell lines visualized with PNA-FISH probe Cy3-OO-$(CCCTAA)_3$ and DAPI. Scale bar: 5µm. **e.** Quantification of chromosomal fusions without telomeric signals in metaphases in (d). 1000 chromosomes analyzed for each condition. **f.** Quantification of chromosomal fusions with telomeric signals in metaphases in (d) with a minimum of 1,000 chromosomes analyzed for each condition.

Supplemental Figure 3. Generation of G7 *Pot1b$^{-/-}$; Tert$^{-/-}$* cell lines and characterization of ALT phenotypes. a. Schematic of *Tert* CRISPR/Cas9 targeting strategy. sgRNA sequence is in bold. PAM sequence is highlighted yellow. **b.** TOPO-TA cloning sequencing results of Tert KO in clones #8 and #9. Yellow highlight indicates the PAM sequence. Red highlights indicate insertions/deletions detected in TOPO-TA sequencing. **c.** TRAP assay of the indicated cell lines. Heat denatured samples were used as negative controls. **d.** Schematic of 2D gel analysis migration patterns. **e.** 2D gel analysis of RsaI and HinfI digested genomic DNA from the indicated cell line and denatured gel hybridization with γ-^{32}P-$(CCCTAA)_4$ telomere probe.

Supplemental Figure 4. Analysis of fragile telomeres and T-SCEs in late-generation *Pot1b$^{-/-}$* cells. a. Representative images of CO-FISH metaphase spreads of the indicated cell lines treated with and without aphidicolin 0.25µM visualized with PNA-FISH probes Cy3-OO-$(CCCTAA)_3$ FAM-OO-$(TTAGGG)_3$ and DAPI. White arrows indicate fragile telomeres. Green arrows indicate T-SCEs. Scale bar: 5µm. **b.** Quantification of fragile telomeres in (a). Data show the mean ± standard deviation from 3 independent experiments with at least 1,000 chromosomes analyzed for each condition per experiment. p-values are shown and generated from one-way ANOVA analysis followed by Tukey's multiple comparison. **c.** Quantification of T-SCEs in (a). Data show the mean ± standard deviation from 3 independent experiments with at least 1,000 chromosomes analyzed for each condition per experiment. p-values are shown and generated from one-way ANOVA analysis followed by Tukey's multiple comparison. **d.** Representative images of combined immunofluorescence TERRA RNA-FISH in the indicated cell lines visualized with antibody against TRF2 (red), PNA-FISH probe Alexa488-OO-$(CCCTAA)_3$ (green), and DAPI (blue). Scale bar: 5µm. **e.** Quantification of nuclei in (d) with ≥5 TERRA/TRF2 colocalizations. U2OS was used as a positive control. 150 nuclei were analyzed for each cell line.

Supplemental Figure 5. Expression of POT1 constructs in late-generation *Pot1b$^{-/-}$* cells. a. Quantification of γ-H2AX TIFs across Pot1b$^{-/-}$ sarcoma generations. 150 nuclei were analyzed for each cell line. **b.** Representative images of γ-H2AX TIFs in independently generated Generation 9 *Pot1b$^{-/-}$; p53$^{-/-}$* cells. Cells were immunostained with γ-H2AX antibody (green), mTRF2 antibody (red) and DAPI to stain nuclei

(blue). Scale bar: 5µm. **c.** Quantification of 150 nuclei with ≥5 γ-H2AX TIFs from (a). **d.** Representative images of immunofluorescence evaluating construct expression in G4 *Pot1b$^{-/-}$* cells. Cells were immunostained with FLAG or MYC antibodies (green), PNA-FISH probe Cy3-OO-$(CCCTAA)_3$ (red) and DAPI to stain nuclei (blue). Scale bars: 5µm. **e.** Quantification of the indicated cell lines with ≥5 colocalizations in (c). Data show the mean ± standard deviation from two independent experiments in which 150 nuclei were analyzed per cell line. **f.** Representative images of p-RPA TIFs in G4 Pot1b$^{-/-}$ cells expressing the indicated constructs. Cells were immunostained with p-RPA antibody (green), PNA-FISH probe Cy3-OO-$(CCCTAA)_3$ (red) and DAPI to stain nuclei (blue). Scale bar: 5µm. **g.** Quantification of the indicated cell lines with ≥5 p-RPA TIFs. 150 nuclei were analyzed per condition.

Supplemental Figure 6. Expression of POT1 chimeric constructs in late-generation *Pot1b$^{-/-}$* cells. a. Representative images of immunofluorescence of G7 *Pot1b$^{-/-}$* cells expressing the indicated constructs. Cells were immunostained with FLAG, MYC or HA antibodies (green), TRF2 antibody (red) and DAPI to stain nuclei (blue). Scale bars: 5µm. **b.** Quantification of the indicated cell lines with ≥5 colocalizations in (a). Data show the mean ± standard deviation from three independent experiments in which 150 nuclei were analyzed per cell line. **c.** Representative images of immunofluorescence of G7 *Pot1b$^{-/-}$* cells expressing the indicated constructs. Cells were immunostained with STN1 antibody (green), TRF2 antibody (red) and DAPI to stain nuclei (blue). Scale bar: 5µm. **d.** Quantification of the indicated cell lines with ≥5 colocalizations in (c). Data show the mean in which 150 nuclei were analyzed per cell line. **e.** Quantification of independent G9 *Pot1b$^{-/-}$; p53$^{-/-}$* cells with ≥5 colocalizations. 150 nuclei were analyzed per cell line. **f.** Representative images of immunofluorescence of independent G9 *Pot1b$^{-/-}$; p53$^{-/-}$* cells expressing the indicated constructs. Cells were immunostained with γ-H2AX antibody (green), PNA-FISH probe Cy3-OO-$(CCCTAA)_3$ (red) and DAPI to stain nuclei (blue). Scale bars: 5µm. **g.** Quantification of the indicated cell lines with ≥5 γ-H2AX TIFs in (f). 150 nuclei were analyzed per cell line.

Supplemental Figure 7. Telomerase recruitment assay POT1b and POT1ba construct expression. a. Representative images of immunofluorescence of G7 Pot1b$^{-/-}$ cells expressing the indicated constructs. Cells were immunostained with FLAG antibody (green), TRF2 antibody (red) and DAPI to stain nuclei (blue). Scale bar: 5µm. **b.** Quantification of the indicated cell lines with ≥5 colocalizations for the expressed construct in (a). Data show the mean ± standard deviation from two independent experiments in which 150 nuclei were analyzed per cell line. **c.** Representative images of immunofluorescence of G3 Pot1b$^{-/-}$ cells expressing the indicated constructs. Cells were immunostained with FLAG antibody (green), TRF2 antibody (red) and DAPI to stain nuclei (blue). Scale bar: 5µm. **d.** Quantification of the indicated cell lines with ≥5 colocalizations for the expressed construct in (c). Data show the mean of 150 nuclei were analyzed per cell line.

Supplementary Figure 8. TERT overexpression in G7 *Pot1b$^{-/-}$; Tert$^{-/-}$* cells. a. Western blot of lysates from G7 *Pot1b$^{+/-}$* and G7 *Pot1b$^{-/-}$; Tert$^{-/-}$* cells expressing the indicated TERT constructs. **b.** TRAP assay of G7 *Pot1b$^{-/-}$; Tert$^{-/-}$* cells expressing the indicated TERT constructs. G7 *Pot1b$^{-/-}$* cells were used as a positive control and heat denatured as a negative control. IC: Internal Control. **c.** Representative images of immunofluorescence G7 *Pot1b$^{+/-}$* and G7 *Pot1b$^{-/-}$; Tert$^{-/-}$* cells expressing the indicated TERT construct. Cells were immunostained with HA antibody (green), TRF2 antibody (red) and DAPI to stain nuclei (blue). Scale bar: 5µm. **d.** Quantification of 150 nuclei from the indicated conditions with ≥3 colocalizations in (c).

Supplementary Figure 9. Characterization of G-overhangs in late-generation *Pot1b$^{-/-}$* cells. a. TRF Southern blot detection of C-strand in native and total telomere length in denatured gel hybridization with γ-^{32}P-$(TTAGGG)_4$ telomere probe. Molecular weight markers as indicated. **b.** Quantification of native signal digested by *E. coli* Exonuclease I shown in Fig. 8a. **c.** Quantification of native signal digested by *E. coli* Exonuclease I shown in Fig. 8b. **d.** TRF Southern blot detection of G-overhang in native and total telomere

length in denatured gel hybridization with γ-^{32}P-$(CCCTAA)_4$ telomere probe of DNA from independently generated Generation 11 *Pot1b*$^{-/-}$*; p53*$^{-/-}$ cells. DNA was treated with and without *E. coli* Exonuclease I in the presence of 150mM KCl or 150mM LiCl as indicated. Numbers indicate relative G-overhang and total telomere signals, with telomere signals set to 1.0 for G7 *Pot1b*$^{+/-}$ without ExoI in 150mM KCl. Molecular weight markers as indicated. **e.** Quantification of native signal digested by *E. coli* Exonuclease I in (d). **f.** TRF Southern blot detection of the G-overhang in native and total telomere length in denatured gel hybridization with γ-^{32}P-$(CCCTAA)_4$ telomere probe of the indicated cell lines expressing the indicated constructs. #8 and #9 indicate two G7 *Pot1b*$^{-/-}$*; Tert*$^{-/-}$ clones generated using CRISPR/Cas9. Numbers indicate relative G-overhang and total telomere signals, with telomere signals set to 1.0 for G7 *Pot1b*$^{-/-}$ cells infected with a vector plasmid without ExoI. Molecular weight markers as indicated. **g.** Quantification of native signal digested by *E. coli* Exonuclease I in (f). **h.** TRF Southern blot detection of the G-overhang in native and total telomere length in denatured gel hybridization with γ-^{32}P-$(CCCTAA)_4$ telomere probe of the G7 *Pot1b*$^{-/-}$ cells expressing the indicated constructs. Numbers indicate relative G-overhang and total telomere signals, with telomere signals set to 1.0 for G7 *Pot1b*$^{-/-}$ cells infected with a vector plasmid without ExoI. Molecular weight markers as indicated. **i.** Quantification of native signal digested by *E. coli* Exonuclease I in (h).
